# Exploring the evolution of *CHS* gene family in plants

**DOI:** 10.3389/fgene.2024.1368358

**Published:** 2024-04-30

**Authors:** Li Yang, Shuai Zhang, Dake Chu, Xumei Wang

**Affiliations:** ^1^ Department of Gastroenterology, The First Affiliated Hospital of Xi’an Jiaotong University, Xi’an, China; ^2^ School of Pharmacy, Xi’an Jiaotong University, Xi’an, China; ^3^ College of Horticulture, Shanxi Agricultural University, Jinzhong, China

**Keywords:** chalcone synthase, flavonoids, phylogeny, gene conservation, synteny network

## Abstract

Chalcone synthase (*CHS*) is a key enzyme that catalyzes the first committed step of flavonoid biosynthetic pathway. It plays a vital role not only in maintaining plant growth and development, but also in regulating plant response to environmental hazards. However, the systematic phylogenomic analysis of *CHS* gene family in a wide range of plant species has not been reported yet. To fill this knowledge gap, a large-scale investigation of *CHS* genes was performed in 178 plant species covering green algae to dicotyledons. A total of 2,011 *CHS* and 293 *CHS-like* genes were identified and phylogenetically divided into four groups, respectively. Gene distribution patterns across the plant kingdom revealed the origin of *CHS* can be traced back to before the rise of algae. The gene length varied largely in different species, while the exon structure was relatively conserved. Selection pressure analysis also indicated the conserved features of *CHS* genes on evolutionary time scales. Moreover, our synteny analysis pinpointed that, besides genome-wide duplication and tandem duplication, lineage specific transposition events also occurred in the evolutionary trajectory of *CHS* gene family. This work provides novel insights into the evolution of *CHS* gene family and may facilitate further research to better understand the regulatory mechanism of traits relating to flavonoid biosynthesis in diverse plants.

## 1 Introduction

Flavonoids are important secondary metabolites due to their biological and pharmacological activities. They are composed of more than 7000 compounds, each containing a C6-C3-C6 carbon skeleton derived from phenylalanine (Wang et al., 2018; Shen et al., 2022). Flavonoids are not only the main components determining the color of flowers and fruits, but also play essential roles in phytohormone transport and plant resistance to various biotic and abiotic stresses (Peer and Murphy, 2007; Mierziak et al., 2014; Iwashina, 2015). Besides, due to their antibacterial, anti-inflammatory, analgesic, and antipyretic properties, flavonoids are considered as natural antioxidants with multiple benefits for the health of human beings (Hoensch and Oertel, 2015).

Chalcone synthase (CHS) is the first enzyme reported to be involved in the initial committed step of flavonoid biosynthetic pathway. It catalyzes the condensation reaction of *p*-coumaroyl-CoA and three malonyl CoA molecules to produce phenyl styrene ketone (chalcone), the precursor of various flavonoid derivatives (Koes et al., 1994; Zhang et al., 2017). CHS enzyme is a member belonging to the plant-specific type III polyketide synthase (PKS) superfamily (Austin and Noel, 2003). It functions as a 40-45 kDa protein homodimers with two independent active sites (Jiang et al., 2008). Members of the CHS family have high similarity in amino acid sequences, with each consisting of two conserved structural domains and a catalytic center composed of four residues, Cys-His-Asn-Phe (Ferrer et al., 1999).

In plants, *CHS* genes have been reported to be involved in a wide range of physiological and biological processes. An earlier research showed that *CitCHS2* had a strong regulatory impact on the accumulation of flavonoids in citrus cell cultures (Moriguchi et al., 1999). This conclusion was undoubtedly correct, as revealed by the functional study of *CHS* gene family members in citrus by Wang et al. (2018), and the correlation analysis between CHS activity and contents of flavonoid pigments by Li et al. (2016). Multiple studies have also documented that the transcript levels of *CHS* genes play important roles in plant response to high temperature stress (Correia et al., 2014; Glagoleva et al., 2019), and light treatment (Zoratti et al., 2014). In addition, the mutation or abnormal expression of *CHS* genes was reported to be associated with male sterility in different plants, such as petunia, radish, and cotton (Napoli et al., 1999; Yang et al., 2008; Kong et al., 2020).

Genome-wide analyses of *CHS* gene family have been performed in various plant species, such as citrus (Wang et al., 2018), soybean (Anguraj Vadivel et al., 2018), *Salvia miltiorrhiza* (Deng et al., 2018), cotton (Kong et al., 2020), eggplant (Wu et al., 2020), *Zostera marina* (Ma et al., 2021), and *Chrysanthemum nankingense* (Zhu et al., 2022). Most of these studies focused on the identification and characterization of *CHS* homologs within a specific species, as well as gene expression profiles in diverse tissues/stages or under different treatments. For example, eight *CHS* genes were identified in *Salvia miltiorrhiza*, and they showed tissue-specific expression patterns and differential transcriptional responses to MeJA treatment (Deng et al., 2018). Despite these advances, the genomic architecture of *CHS* family in the evolutionary trajectories has not yet been investigated.

With the availability of numerous sequenced genomes and the development of bioinformatic tools, such as the synteny network approach for large-scale synteny computation by Zhao and Schranz (2017), large-scale phylogenomic analyses (combined phylogenetic and synteny analysis) are widely used in studying the genetics and the evolution of complex gene families (Zhao et al., 2017; Kerstens et al., 2020). To gain insights into how *CHS* gene family evolved, we performed a comprehensive phylogenomic analysis of *CHS* genes from 178 plant species covering green algae to dicotyledons. Our results revealed the early origin of this gene family across the plant kingdom. Selection pressure analysis pinpointed out the conserved features of *CHS* genes on evolutionary time scales. In addition, the phylogeny, gene structure, protein characteristics, and synteny network were systematically investigated. This work broadens our understanding of the evolution of *CHS* gene family and provides compelling opportunities for further functional studies on flavonoid biosynthesis.

## 2 Materials and methods

### 2.1 Identification of *CHS* family members

A set of 178 plant genomes, basically from Pancaldi et al. (2022), was used for analysis in this study (Supplementary Table S1). Information of species taxonomic classification was obtained from Angiosperm Phylogeny Website (APG) and NCBI databases (Leebens-Mack et al., 2019). Species tree was constructed by ETE 3.1.1 and subsequently visualized in iTOL v5 (Huerta-Cepas et al., 2016; Letunic and Bork, 2021).

Two different methods were employed to identify *CHS* family members. Four amino acid sequences of *Arabidopsis thaliana CHS* genes were firstly used as queries to search against the protein databases of 178 plant genomes using BLAST 2.14.0 with an e-value of 1e-2 (Camacho et al., 2009). Obtained protein sequences were then aligned using MAFFT v7 (Katoh et al., 2019; Supplementary MaterialS1, 2), followed by gap filtering in trimAl 1.2rev59 with parameters gt 0.8, st 0.001, and cons 60 (Capella-Gutiérrez et al., 2009). Filtered multiple sequence alignment (MSA) was finally used to construct maximum likelihood tree in FastTree 2.1.11 (Price et al., 2010). Tree branches containing query sequences and conforming to evolutionary relationships were retained, and gene hits on the branches were considered as candidate homologs. In parallel, the Hidden Markov Model (HMM) profiles of Chal_sti_synt_C (PF00195) and Chal_sti_synt_N (PF02797) domains were downloaded from Pfam database (http://pfam.xfam.org/), and were used to construct CHS HMM using hmmbuild implemented in HMMER 3.3.2 (Finn et al., 2011). The specific CHS HMM files were subsequently employed as inputs to search against aforementioned protein databases using hmmsearch. The resulting outputs were then mutually verified with the results of BLAST search. Finally, only hits that contain both Chal_sti_synt_C and Chal_sti_synt_N domains were designated as true *CHS* homologs, while those containing either of two domains were considered as *CHS-like* homologs.

### 2.2 Phylogenomic analysis and gene classification

Amino acid sequences of 2,011 *CHS* and 293 *CHS-like* genes were identified and used for phylogeny analysis and classification. Gene names were represented by adding abbreviated species prefix to the original names. The evolutionary trees of *CHS* and *CHS-like* genes were rooted in *XP_005651931.1* and *XP_005650884.1*, homologs from *Coccomyxa subellipsoidea*, the early-branching lineage of Chlorophyta, respectively. MSA was obtained by using MAFFT v7.520 with default settings (Katoh et al., 2019), followed by the filtration of gap columns using trimAl 1.2rev59 (Capella-Gutiérrez et al., 2009). Subsequently, IQ-TREE v2.2.2.9 was employed to construct maximum-likelihood tree with the parameters of model MFP and bootstrap replicates 1,000 (Nguyen et al., 2015). Webtool iTOL v5 was used for the final visualization of phylogeny trees (Letunic and Bork, 2021).

### 2.3 Gene structure and protein characteristic analysis

Gene structure information of *CHS*/*CHS-like* genes in every species was parsed from the corresponding GFF3 files using an in-house Perl script. Gene length, CDS number, and CDS length were indicated by mean values when multiple gene copies were presented in one species. Protein characteristics, including molecular weight (MW), isoelectric point (pI), and hydropathicity (GRAVY), were predicted using online website (http://www.detaibio.com/sms2/).

### 2.4 Selection pressure analysis

Homologous gene pairs were firstly identified in each representative species using reciprocal BLASTP with the threshold of identity >50%. Nucleotide and amino acid sequences per genome were aligned by MAFFT v7.520 (Katoh et al., 2019), and the ratio of nonsynonymous substitutions (Ka) to synonymous substitutions (Ks) of each homologous pair was estimated by KaKs_Calculator implemented in ParaAT2.0 (Wang et al., 2010; Zhang et al., 2012). Ka/Ks values <1 represents negative or purifying selection, while Ka/Ks values >1 is regarded as positive selection.

### 2.5 Synteny network construction and clustering

Pair-wise comparisons of protein sequences from 178 plant genomes were conducted by software Diamond v2.0.11.149 (Buchfink et al., 2015). The top five hits of each genome were recruited as inputs to detect syntenic blocks using MCScanX, with a minimum match size of three and a maximum gap of 25 (Wang et al., 2012). The outputs formed a synteny network across 178 genomes, among which nodes represent genes and edges indicate syntenic relationships between genes. Edges with two *CHS*/*CHS-like* genes were extracted by Shell script and designated as *CHS*/*CHS-like* synteny network, which was graphically represented in Gephi v0.9.2 (Bastian et al., 2009). Synteny clusters were identified by executing the Infomap function in R package igraph (Rosvall and Bergstrom, 2008), and those containing at least three nodes were retained and visualized in Cytoscape v3.8.2 (Shannon et al., 2003). Profiling of species and node numbers within each cluster was investigated using an in-house R script. This was followed by cluster dissimilarity computation using Jaccard method and hierarchical clustering using ward. D (Dixon, 2003; Kolde, 2012). Collinear connections between gene nodes were graphically shown in evolutionary trees using iTOL v5 (Letunic and Bork, 2021).

## 3 Results

### 3.1 Genome-wide identification of *CHS*/*CHS-like* genes in plants

**FIGURE 1:**
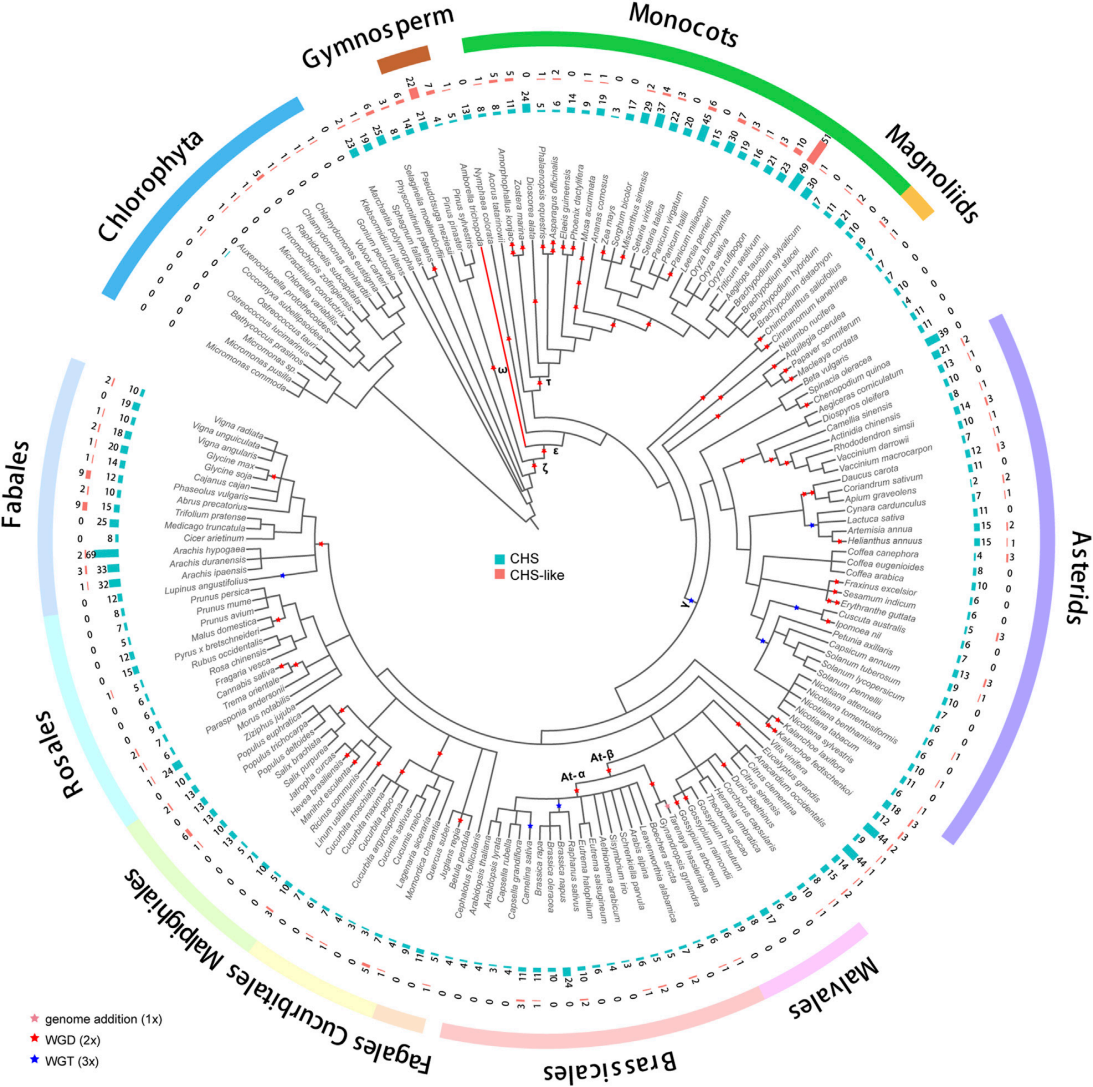
Distribution of *CHS* and *CHS-like* genes across 178 plant genomes. The genome duplication events are inferred from earlier studies (Zhao et al., 2017; Gao et al., 2020; Kerstens et al., 2020; Liu et al., 2022; Ma et al., 2022; Hoang et al., 2023). Pink, red, and blue stars represent known genome addition, WGD, and WGT events, respectively. Clades of species belonging to Fabales, Rosales, Malpighiales, Cucurbitales, Fagales, Brassicales, Malvales, Asterids, Magnoliids, Monocots, Gymnosperm, and Chlorophyta are color-coded. The basal angiosperm *Amborella trichopoda* is indicated with a red branch. The bar plots from inner to outer layers display the copy numbers of *CHS* and *CHS-like* genes, respectively.

A group of 178 genomes from different plant species (Supplementary Table S1) (Pancaldi et al., 2022), with a range from Chlorophyta to flowering plants, was collected for genome-wide identification of *CHS* gene family. A total of 2,011 *CHS* homologous genes was detected in 162 genomes (Figure 1; Supplementary Tables S2-S4), with 12.4 members per genome on average. The coefficient of variation (CV) of the *CHS* copy number was 80%, much higher than what is found in the conserved gene family such as *CesA* (40%) (Pancaldi et al., 2022). This difference could be largely explained by diverse functional characteristics. *CHS* genes, playing a role in the biosynthesis of secondary metabolites (Ma et al., 2021), tend to have higher variability as a result of evolutionary adaption to abiotic and biotic stresses. In addition, we also identified 293 *CHS-like* genes in 98 genomes (∼3 per genome, CV = 189%, Figure 1; Supplementary Tables S2-S4). The copy number of *CHS*/*CHS-like* genes was found to correlate with the ploidy level (cor = 0.45/0.43, *p* < 0.001 for both, Supplementary Figure S1), but not with the number of genome duplications in each species (cor = 0.08/0.03, *p* > 0.01 for both, Supplementary Figure S2). This result implies that other factors, such as local gene duplications and gene losses, also impact the size of *CHS* and *CHS-like* gene families during evolution.

### 3.2 Phylogenetic analysis and classification of *CHS*/*CHS-like* genes

**FIGURE 2:**
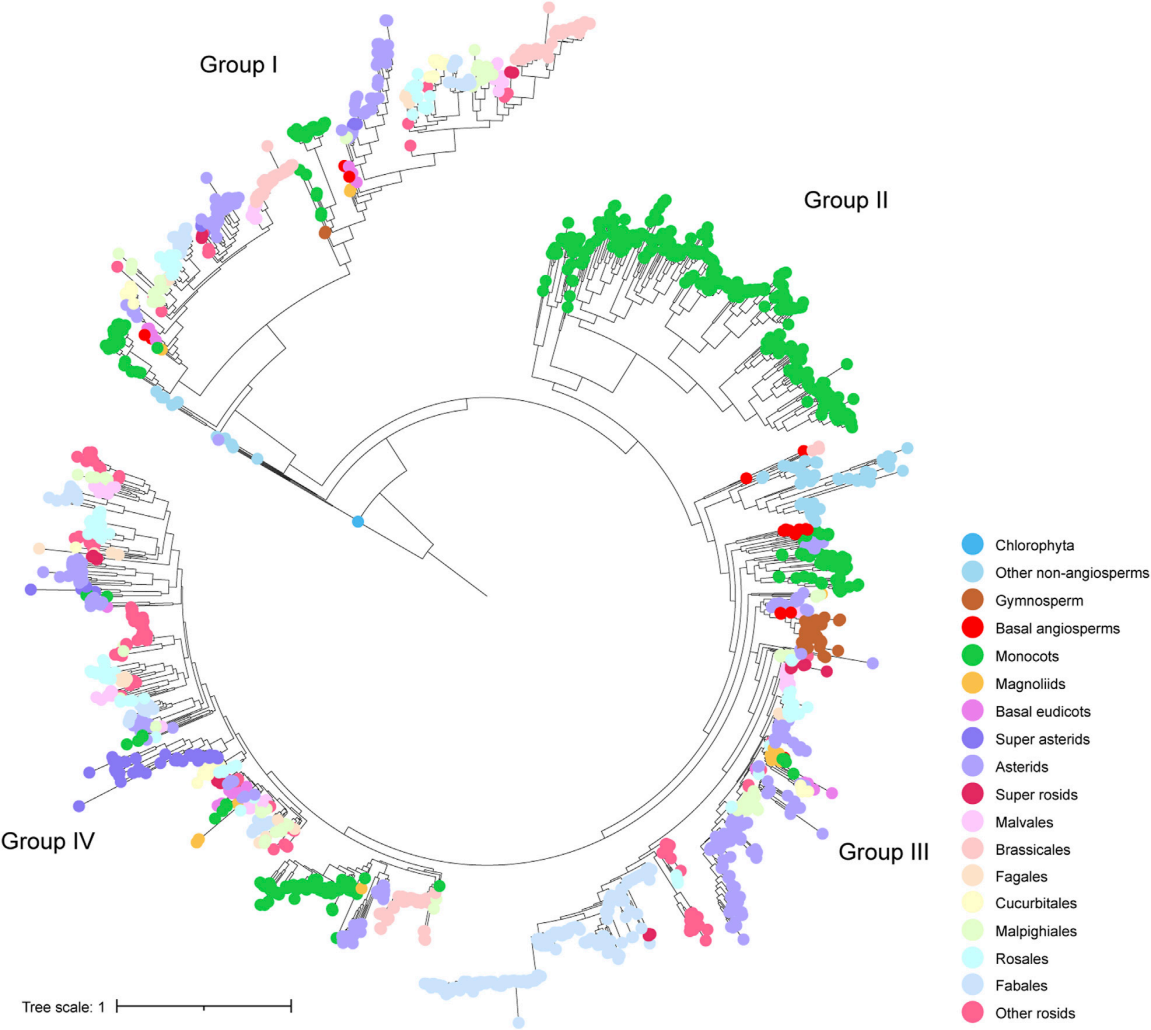
Phylogenetic tree of identified *CHS* genes. Four subgroups were indicated by group labels (I - IV). Colorful dots at the tips of leaves represent different clades of species to which different genes belong.

We performed phylogenetic analysis to investigate the evolutionary characteristics of *CHS* and *CHS-like* genes. The distribution of *CHS* genes across 162 plant genomes showed that *CHS* appeared as early as the green algal phase, suggesting that the origin of *CHS* can be traced back to before the rise of algae (Figure 1). The 2,011 *CHS* homologs were phylogenetically categorized into four groups (Figure 2; Supplementary Figure S3). Group I possessed three out of four query *AtCHS* sequences, and contained *CHS* genes covering Chlorophyta *Coccomyxa subellipsoidea*, gymnosperm, monocots, magnoliids, and a wide spectrum of eudicot clades, including asterids, Malvales, Brassicales, Cucurbitales, Malpighiales, Rosales, and Fabales. Group II was only confined to monocots, with high copy numbers in Poaceae, especially in diploid *Miscanthus sinensis*. Group III had the most *CHS* homologs from diverse plant species, including bryophytes, gymnosperm, monocots, magnoliids, and most eudicot orders. Here, we observed that *CHS* genes were extensively expanded in asterids (145) and Fabales (229), and remarkably low in Brassicales (3). Group IV was angiosperm-specific, with *CHS* genes from monocots, magnoliids, and eudicots, and included another query *AtCHS* sequence. The result of a relatively simple species composition in group IV implies a later origin of the genes within this group compared to those of group I and III.

Phylogenetic analysis on *CHS-like* genes also provided evidence that 293 *CHS-like* genes were clustered into four groups (Supplementary Figure S4). Group I contained 33 *CHS-like* genes from species covering Chlorophyta, lycophytes, bryophytes, and few angiosperms. Group III was mainly composed of *CHS-like* homologs from monocots, while group II and IV possessed a wide range of species, i.e., from lycophytes, bryophytes, gymnosperm, monocots, magnoliids, to dicotyledonous plants such as Fabaceae.

### 3.3 Features of gene structure, protein characteristic, and selection pressure estimation

**FIGURE 3:**
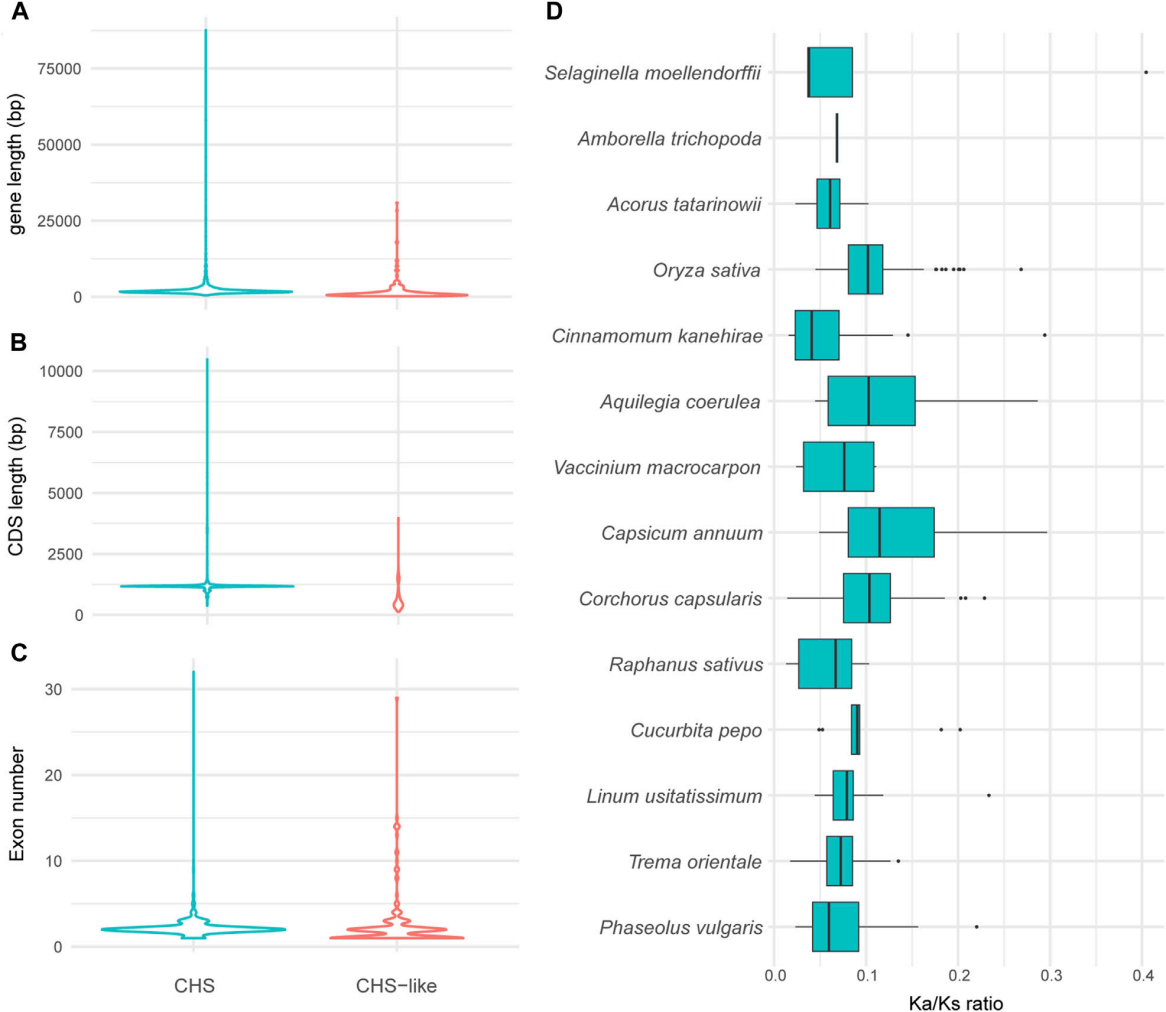
The variation of gene structures and Ka/Ks values of *CHS* and/or *CHS-like* genes. **(A–C)** Graphical display of gene length **(A)**, CDS length **(B)**, and exon number **(C)** of all analyzed plant genomes. **(D)**, Ka/Ks values of *CHS* in 14 representative species. The Ka/Ks of *Coccomyxa subellipsoidea* was excluded due to only one gene copy of *CHS*.

To exploit how gene structure of *CHS*/*CHS*-like genes changed during the time scale of evolution, we performed exon-intron structure analysis for all obtained genes. The *CHS* homologs have slightly longer gene lengths than *CHS-like* genes (Figure 3A), while the CDS lengths of *CHS* were about twice as long as what were observed for *CHS-like* (Figure 3B). However, there was no significant difference in exon number between these two types of genes (Figure 3C).

We next used 15 out of 178 genomes to systematically study the exon-intron structures of *CHS* and *CHS-like* genes in species level (Table 1). Generally, *CHS* genes varied largely in gene lengths across different species (CV = 47%), while their CDS lengths were relatively conserved (CV = 15.0%). This variation may be caused by the presence of introns or transposons, since most *CHS* genes have more than two exons. When looking into *CHS-like* genes, both gene lengths and CDS lengths differed greatly, with the CV of 95.3% and 66.6%, respectively. We speculate that this may be due to the fusion of other protein domains with Chal_sti_synt_N/C domains contained in *CHS-like* sequences.

**TABLE 1:** Structural characteristics of *CHS* and *CHS-like* genes in 15 representative species.

Abbreviation	Species	Gene number		Average gene length		Average exon number		Average CDS length	
		*CHS*	*CHS-like*	*CHS*	*CHS-like*	*CHS*	*CHS-like*	*CHS*	*CHS-like*
DP	*Coccomyxa subellipsoidea*	1	1	5,653	4541	13	9	1,665	1,323
LQ	*Selaginella moellendorffii*	8	3	1,443	2,405	2.125	1.67	1,160	1,498
BD	*Amborella trichopoda*	5	1	3,142	2,441	3.2	1	1,101	1,410
PU	*Acorus tatarinowii*	8	1	2,103	2,577	3	2	1,188	1,098
HN	*Oryza sativa*	21	3	2,805	501	2.05	1.67	1,210	407
DC	*Cinnamomum kanehirae*	7	1	2,776	393	2.29	1	1,186	393
AF	*Aquilegia coerulea*	7	0	1725	−^1^	2	-	1,150	-
PY	*Vaccinium macrocarpon*	7	1	1886	303	1.86	1	1,138	303
BZ	*Capsicum annuum*	9	1	2,360	1,696	2.11	2.00	1,176	798
CL	*Corchorus capsularis*	10	1	1987	394	2.9	2.00	1,265	222
LF	*Raphanus sativus*	10	2	1,570	1,023	2.1	2.00	1,142	486
DH	*Cucurbita pepo*	7	1	2077	691	3.14	2.00	1,666	483
FZ	*Linum usitatissimum*	10	3	1728	506	2.1	1.67	1,153	442
MI	*Trema orientale*	7	2	1,274	436	1.71	2.00	1,070	264
IZ	*Phaseolus vulgaris*	12	9	1,625	536	2	1.56	1,129	423

'-, gene absence.

We then focused on protein characteristics of *CHS* and *CHS-like* genes (Supplementary Table S5), including amino acid length (aa), isoelectric point (pI), molecular weight (MW), and hydrophilia preference (GRAVY). In general, genes belonging to *CHS* have longer protein sequences, with 397 aa on average. This is more than twice as long as what was found for CHS-like, which displays an average of just 199 aa. This tendency is exactly consistent with the result of their MW values, with the average weight of 43.4 and 22.0 kDa, respectively. Moreover, slight variations were detected between CHS and CHS-like proteins in terms of pI and GRAVY, with all sequences around seven and -0.1, respectively.

To estimate the selection pressure of *CHS/CHS-like* genes, the ratio of nonsynonymous substitutions (Ka) to synonymous substitutions (Ks) of each homologous pair in 15 representative plant species was calculated using ParaAT2.0 software (Zhang et al., 2012). The Ka/Ks values were not shown for all *CHS-like* genes, as well as *CHS* gene from *Coccomyxa subellipsoidea*, since only one or zero gene copies were detected in most analyzed species. The Ka/Ks ratios of all retained *CHS* homologous pairs were found to be lower than 1, revealing that *CHS* genes have undergone negative selection (Figure 3D). This result indicates the conserved feature of *CHS* genes on evolutionary time scales, and also reflects the importance of secondary metabolite biosynthesis in plant species.

### 3.4 Gene duplication and synteny network analysis

To explore the syntenic conservation of *CHS* and *CHS-like* genes, we performed phylogenomic synteny network analysis based on the genomic contexts in each genome. About 65.0% of the *CHS* (1,307 out of 2,011) genes were present in *CHS* synteny network with 22,946 connections (Supplementary Table S6). This is a fairly low percentage, approximately 15%–24% lower than those found in synteny studies working on highly conserved gene families, such as *MADS-box* and *CesA*/*Csl* (Zhao et al., 2017; Pancaldi et al., 2022). This result suggests that the synteny of *CHS* genes playing a role in secondary metabolite biosynthesis is much weaker than that of genes involved in developmental process. However, only 8.9% of the *CHS-like* genes (26 out of 293) were contained in *CHS-like* synteny network with 27 connections in total (Supplementary Table S6).

**FIGURE 4:**
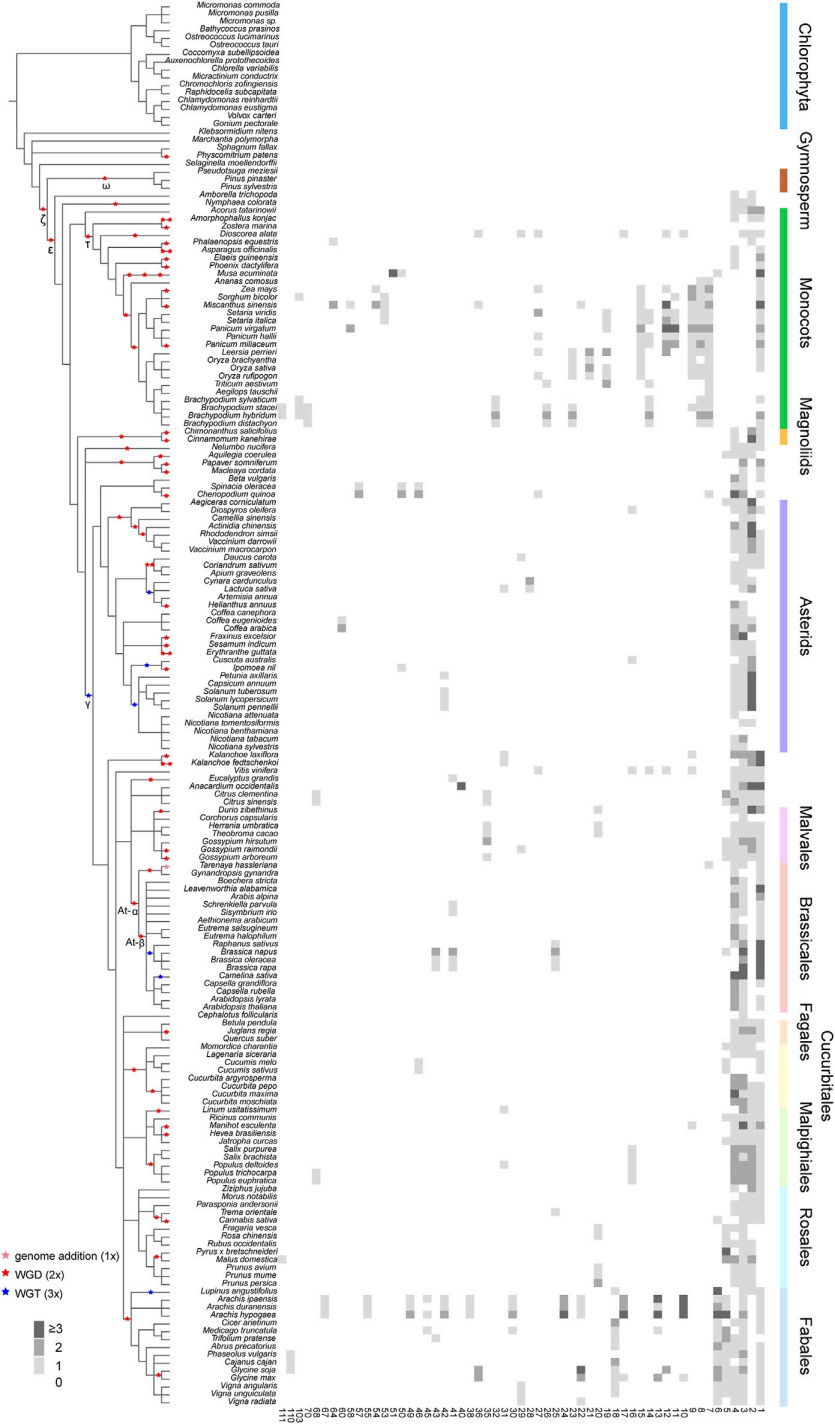
Phylogenomic profiling of CHS syntenic clusters. The genome duplication events are inferred from earlier studies (Zhao et al., 2017; Gao et al., 2020; Kerstens et al., 2020; Liu et al., 2022; Ma et al., 2022; Hoang et al., 2023). Pink, red, and blue stars represent known genome addition, WGD, and WGT events, respectively. Clades of species belonging to Fabales, Rosales, Malpighiales, Cucurbitales, Fagales, Brassicales, Malvales, Asterids, Magnoliids, Monocots, Gymnosperm, and Chlorophyta are color-coded. Rows and columns represent species and clusters, respectively. Gene numbers per species within each cluster is indicated by a grey gradient.

**FIGURE 5:**
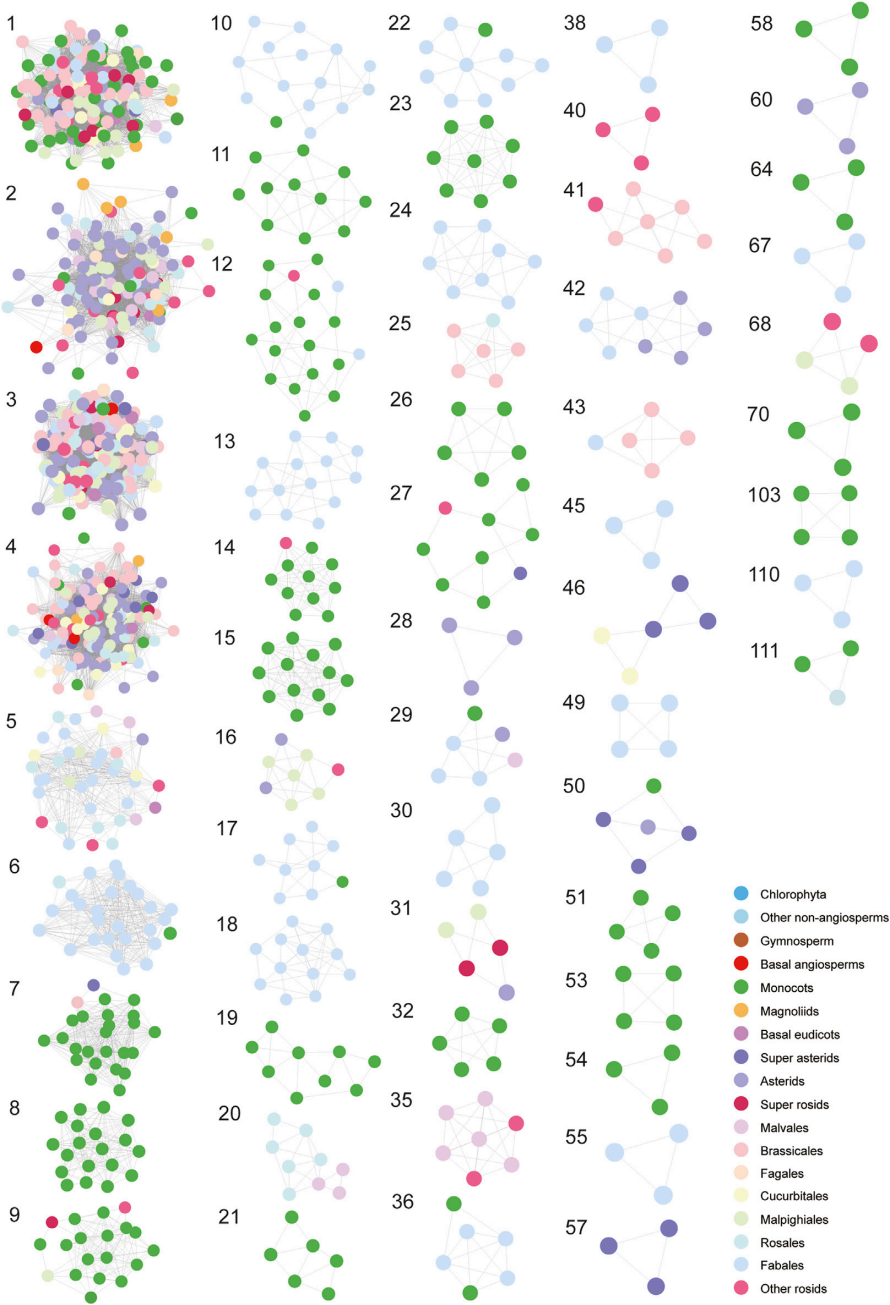
Visualization of each syntenic cluster in accordance with the order of clusters in Figure 4. The colorful nodes (syntelogs) represent different clades of species to which different genes belong.

For a next step, by decomposing synteny networks into communities of closely related gene pairs, a total of 57 CHS and 4 CHS-like clusters were identified with 992 and 15 syntelogs, respectively (Figures 4, 5; Supplementary Figures S5-S6; Supplementary Table S7). The phylogenomic profiles of CHS and CHS-like synteny clusters are remarkably different. Four large and dense CHS clusters (1-4) were highly conserved, consisting of *CHS* syntelogs from basal angiosperms to dicotyledon plants (Figures 4, 5; Supplementary Table S7). Besides, several smaller clusters (6-7, 9-10, 12, 14, 17, 22, 27, 29, 36, 50 and 111) were also relatively conserved across angiosperm species. Clusters 5, 16, 31, 42, and 46 contained *CHS* genes of different genomes from diverse clades of eudicots. Moreover, multiple groups of genes belonging to specific plant branches were found with different sizes. For example, rosids, monocots, and Fabales were discovered in a multitude of clusters (6-15), while asterids and super asterids were only represented in a few clusters (1-2; Figures 4, 5; Supplementary Table S7). Next to the findings of *CHS*, we also parsed the species composition of *CHS-like* synteny network (Supplementary Figure S6A). Two of the four identified synteny clusters contained *CHS-like* genes of genomes covering magnoliids and eudicots (1, 5), while the other two clusters were specific to Chlorophyta (4) and Malpighiales (2; Supplementary Figure S6A; Supplementary Table S7).

**FIGURE 6:**
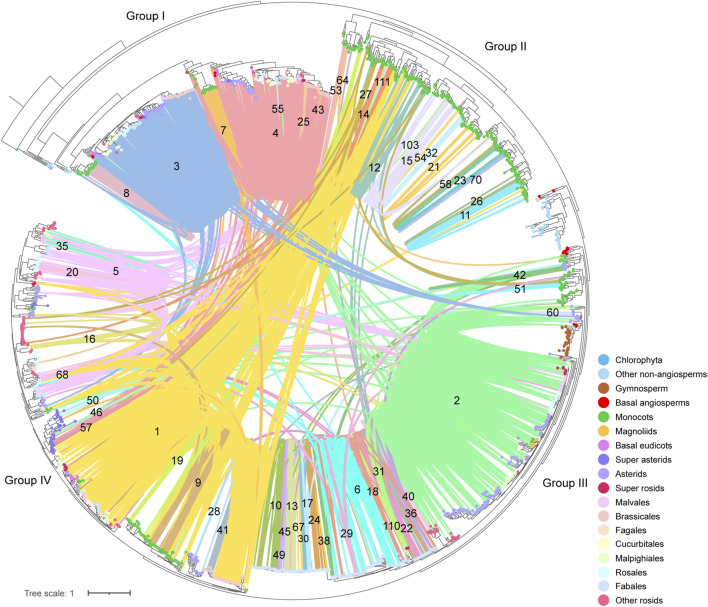
Phylogenetic classification and syntenic relationships of *CHS* genes. The number located on the syntenic line indicates the number of the synteny cluster. The colorful dots on the tips of leaves represent different clades of species to which different genes belong.

To deep into the genomic organization of *CHS* genes and gain insights into their relevance to the evolution, we combined the syntenic connections within each of the clusters with the phylogenetic trees mentioned earlier (Figure 6; Supplementary Figure S6D). In general, puny consistency was found between syntenic conservation of gene architecture and phylogenetic classification of *CHS* genes, especially for genes belonging to group II and group IV. One largest cluster (1), comprising 5,412 connections, widely spanned these two groups. Further analysis showed that this cluster covered 14.2% of the syntenic *CHS* genes and 59.0% of the plant species included in this study. These results indicate that genomic contexts of group II and group IV experienced severe genomic rearrangements. The other two groups were relatively independent from each other. Specifically, group I was mainly sub-organized by two clusters (3, 4), and group III spanned one of the largest clusters (2). Except these several dense clusters, a number of syntenic connections within multiple small clusters were found to spread across different *CHS* groups. These could be resulted from the background noise of synteny analysis according to Pancaldi et al. (2022). In addition, syntelogs from several monocots-specific clusters (8, 32, 51, 53, 54, and 70) were found to phylogenetically form into monophyletic clades, as well as those belonging to Fabales (24, 49, 55, 67, and 110), Sapindales (40), asterids (60), and super asterids (57). These results indicate that *CHS* genes have undergone abundant ancient transposition activities within these categories.

## 4 Discussion

The development of bioinformatics and the completion of various sequenced genomes provide us easier ways to study genetic variability and evolution. CHS is an essential enzyme involved in the production of flavonoid derivatives and plays an important role in biological processes related to plant growth and development (Kong et al., 2020). *CHS* family has been reported in a large number of plant species, such as soybean, cotton, and eggplant among many others (Anguraj Vadivel et al., 2018; Kong et al., 2020; Wu et al., 2020). However, gene identification in distantly related genomes is not always easy to perform. In this study, we employed two independent approaches, blast similarity search combined with phylogenetic analysis and Pfam domain search, to identify *CHS*/*CHS-like* homologs from 178 plant species. Compared with several related studies that only relied on a blast or Pfam threshold, the approach adopted in this study based on both phylogenetic relationships and domain presence is more reasonable and reliable. Most of the hits obtained by blast/phylogeny and Pfam were overlapping with each other, except for a small number of specific hits (data not shown), which were subsequently determined manually. This finally leads to the identification of 2,011 *CHS* and 293 *CHS-like* homologs from 162 to 98 plant genomes, respectively (Supplementary Tables S2-S4). Slight differences in *CHS* gene copies were found in comparison with former studies, such as six less in rice and three more in maize (Han et al., 2016; Han et al., 2017), which may be due to the update of genome versions or genome annotations.

Few studies have explored the genomic architecture of this gene family across the evolutionary time scales. A comparable phylogeny study by Xie et al. (2016) using a limited number of species showed that *CHS* genes were first found in bryophytes. However, the presence of *CHS* gene in *Coccomyxa subellipsoidea* (Supplementary Table S2), provides evidence that the origin of this gene can be traced back to early Chlorophyta in this current study. This result implies that limited taxon sampling cannot obtain a complete picture of gene family evolution. In addition, the 2,011 *CHS* homologs were phylogenetically clustered into four groups (Figure 2). This is consistent with the classification by Zhu et al. (2022), who studied the phylogeny of *CHS* genes within several plant species, but different from the categorization found in other related studies (Kong et al., 2020; Wu et al., 2020; Ma et al., 2021). Ruling out that this difference is due to different taxon sampling, it would suggest that the division of gene families should take into account not only phylogeny classification, but also other results such as gene function, structure variations, and expression patterns.

As previous studies pinpoint out that most *CHS* genes contain two exons and one intron (Kong et al., 2020; Zhu et al., 2022), the same does also hold for *CHS* homologs identified in our work, with a few exceptions that may be caused by assembly errors (Figure 3). However, the number of exons varied considerably among *CHS-like* genes, indicating potential function divergence possibly allowed by relaxed selection on redundant genes. In addition, the Ka/Ks ratios of *CHS* gene pairs in 14 representative plant species were less than 1, which was identical to those found in former studies (Anguraj Vadivel et al., 2018; Ma et al., 2021; Zhu et al., 2022). One exception was found in *Gossypium barbadense* that was not included in our study, with six pairs of duplicated genes having Ka/Ks ratios greater than 1, indicating the presence of positive selection (Kong et al., 2020). If this is true, whether this pattern is also present in other species is not clear. Future work could address this question by investigating *CHS* genes from more related plant genomes. Several reports also revealed the diverse expression patterns of *CHS* genes in different tissues and developmental stages (Wang et al., 2018; Kong et al., 2020). This may raise more questions that could be explored by future studies. How is this expression differentiation evolved from lower plants to higher plants? How is it associated with phenotype evolution?

## 5 Conclusion

*CHS* is regarded as an important enzyme involved in the production of flavonoid derivatives and plays a role in various physiological and biological processes. In this study, we performed a phylogenomic analysis of *CHS* gene family using 178 genomes with a range from Chlorophyta to flowering plants. Our results revealed the early origin of *CHS* and *CHS-like* genes, that is, before the rise of algae. The conservation in gene structure and the negative selection of *CHS* genes indicate the conserved nature of flavonoid pathway, which also reflects the functional importance of flavonoid biosynthesis in plants. The synteny network analysis of *CHS* gene family also pinpointed both conservation and lineage-specific patterns. These findings provide novel insights into the evolutionary history of *CHS* gene family.

## Data Availability

The original contributions presented in the study are included in the article/Supplementary Material, further inquiries can be directed to the corresponding authors.
