# Supplementary figures and images for: Exploring the evolution of CHS gene family in plants
Source: Front Genet. 2024 Apr 30;15:1368358. doi: 10.3389/fgene.2024.1368358 (PMC11091334; doi:10.3389/fgene.2024.1368358)

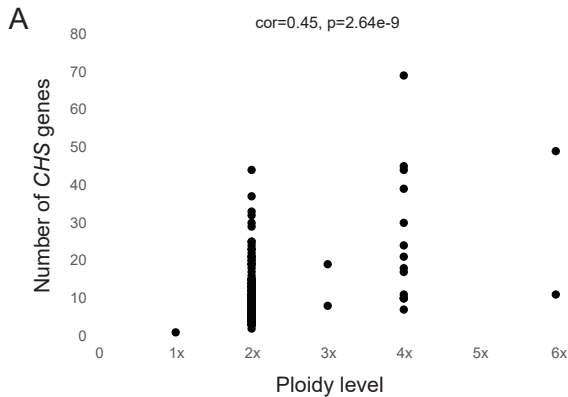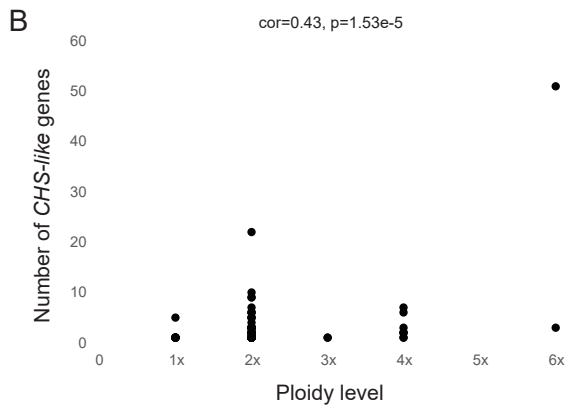

Supplement: Supplementary file 1 [file DataSheet1.ZIP › Fig.S1.pdf]

A

cor=0.08, p=0.32

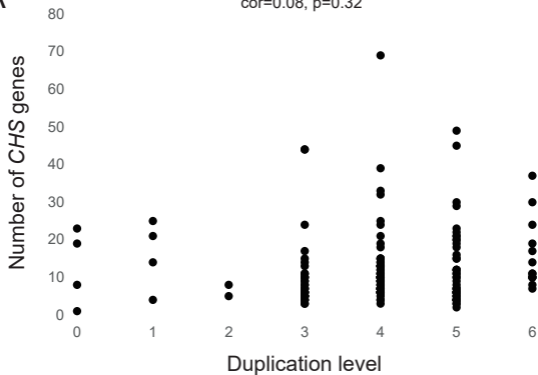

B

cor=0.03, p=0.76

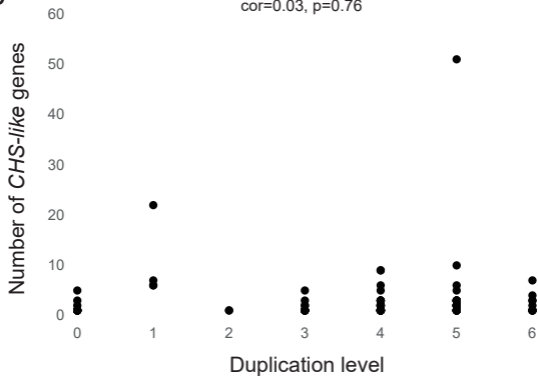

Supplement: Supplementary file 1 [file DataSheet1.ZIP › Fig.S2.pdf]

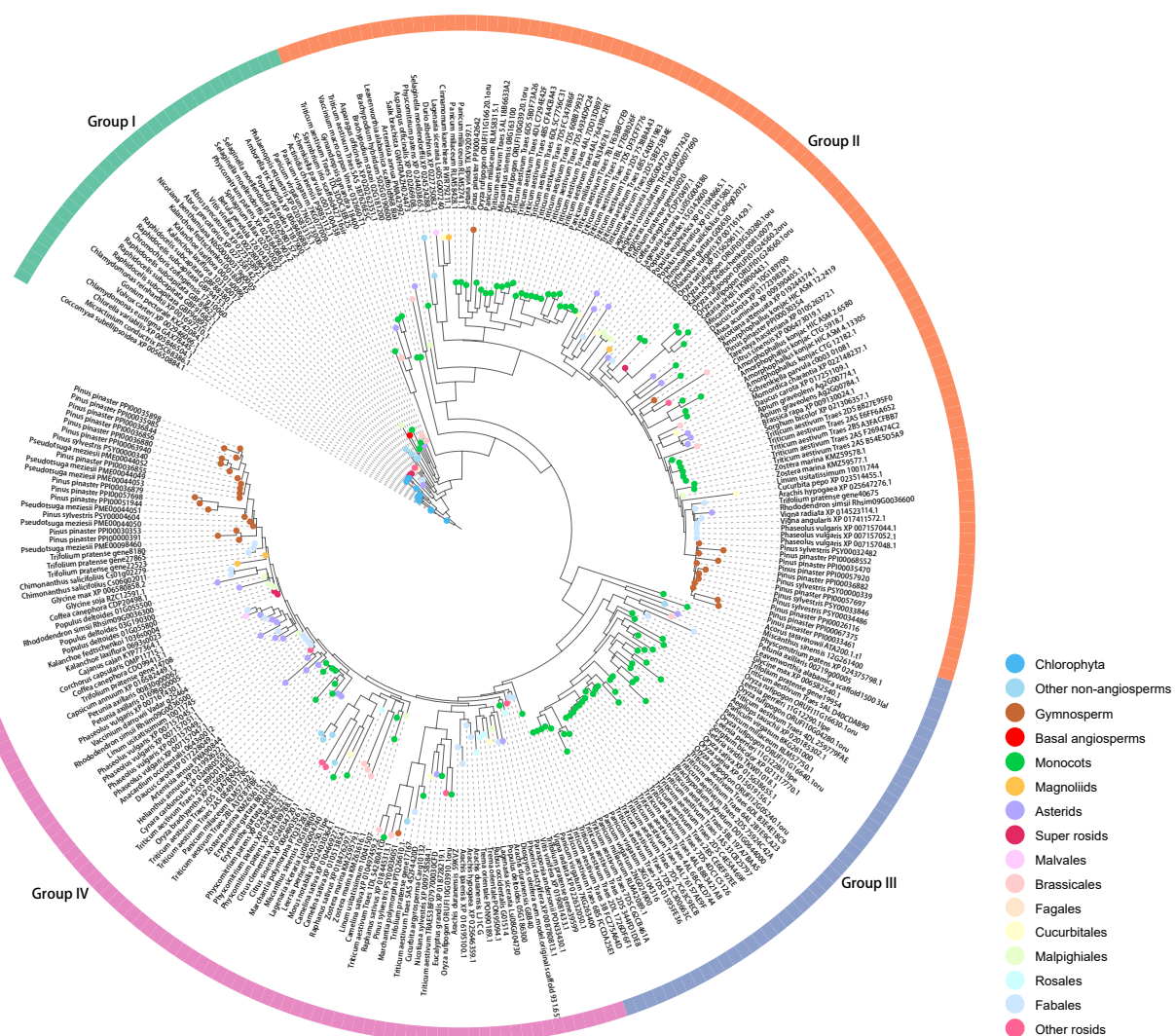

Supplement: Supplementary file 1 [file DataSheet1.ZIP › Fig.S4.pdf]

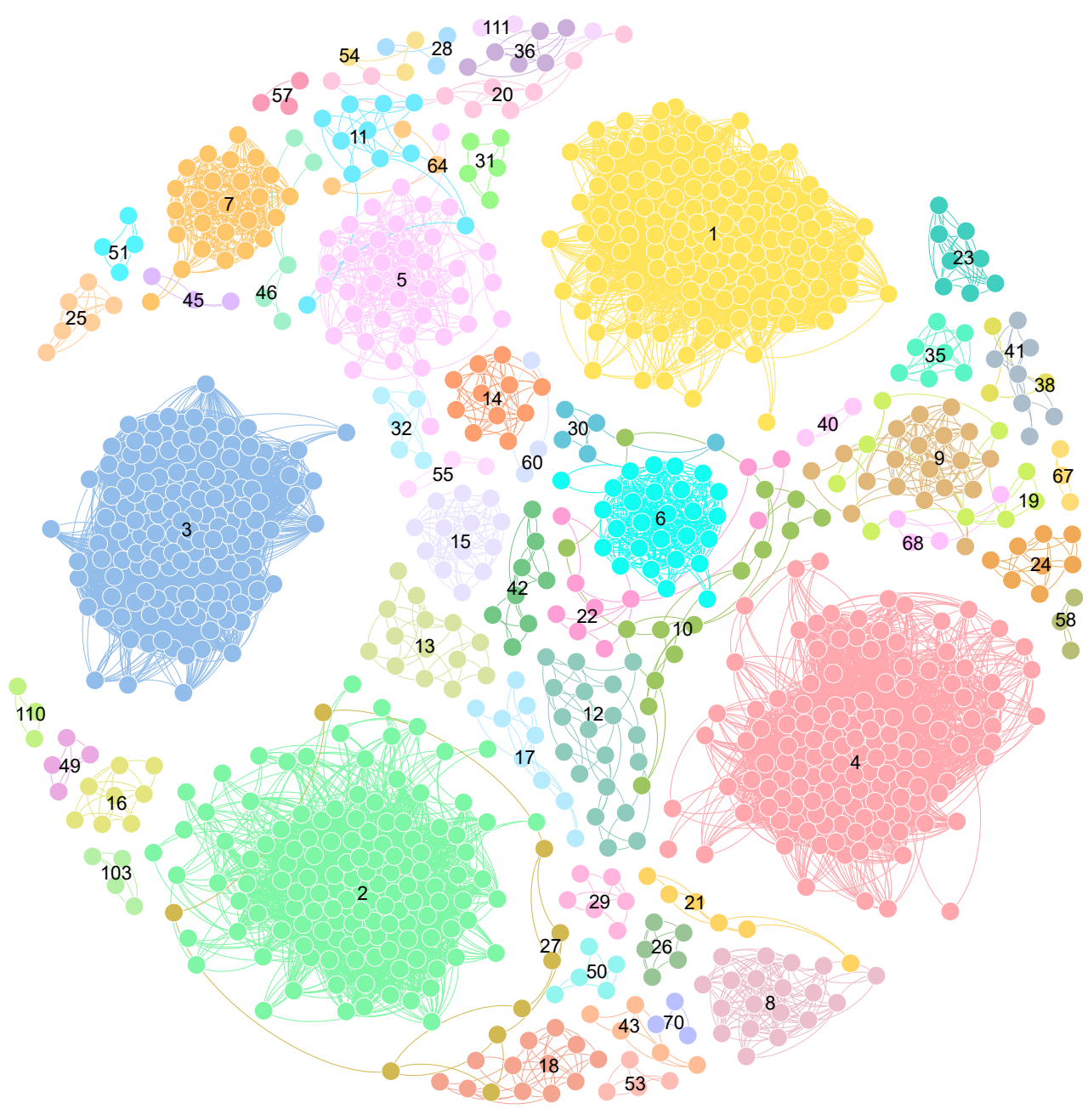

Supplement: Supplementary file 1 [file DataSheet1.ZIP › Fig.S5.pdf]

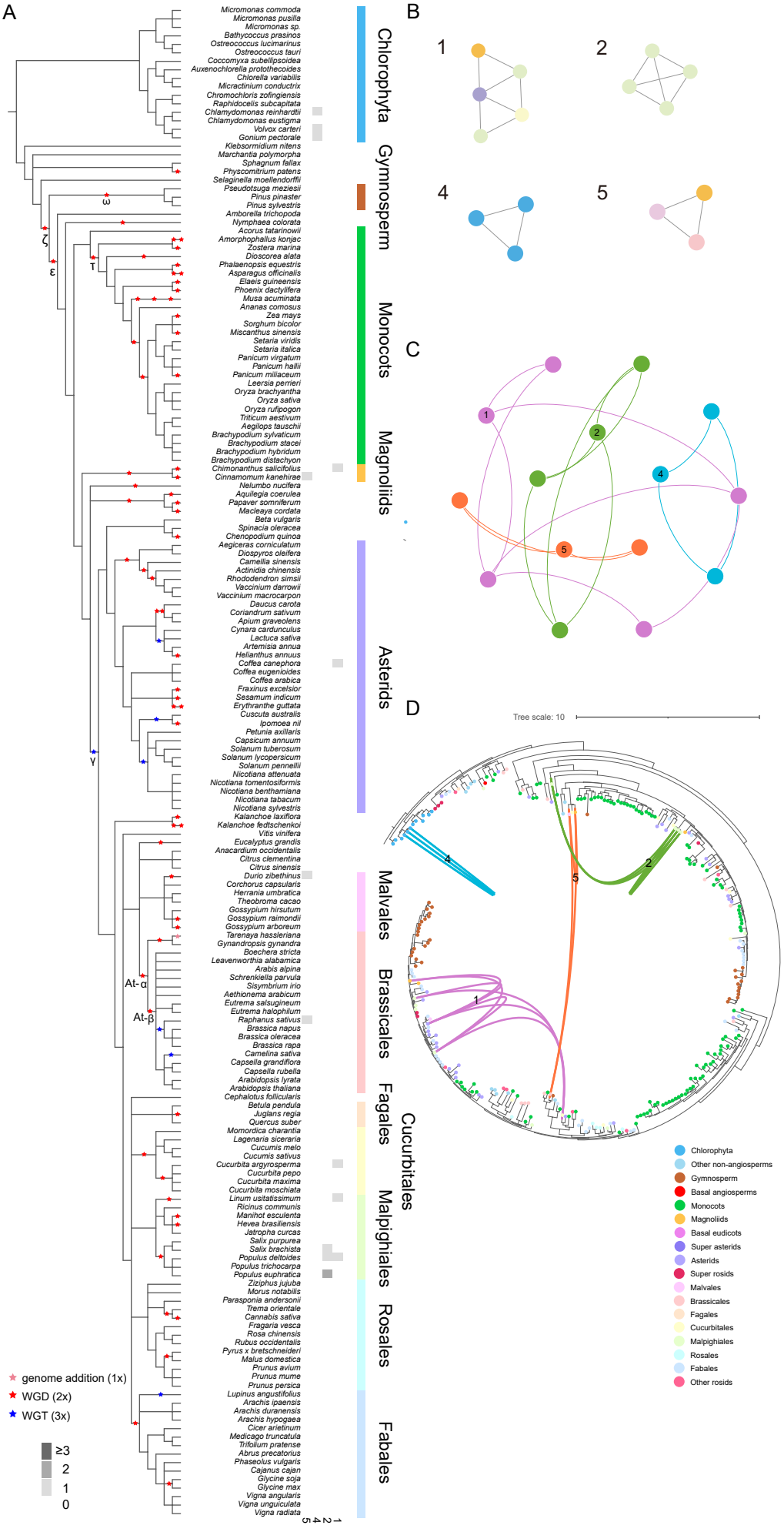

Supplement: Supplementary file 1 [file DataSheet1.ZIP › Fig.S6.pdf]
