# Supplementary material for: Exploring the evolution of CHS gene family in plants
Source: Front Genet. 2024 Apr 30;15:1368358. doi: 10.3389/fgene.2024.1368358 (PMC11091334; doi:10.3389/fgene.2024.1368358)
Supplement: Supplementary file 1 [file Fig.S3.pdf › Fig.S3.pdf]

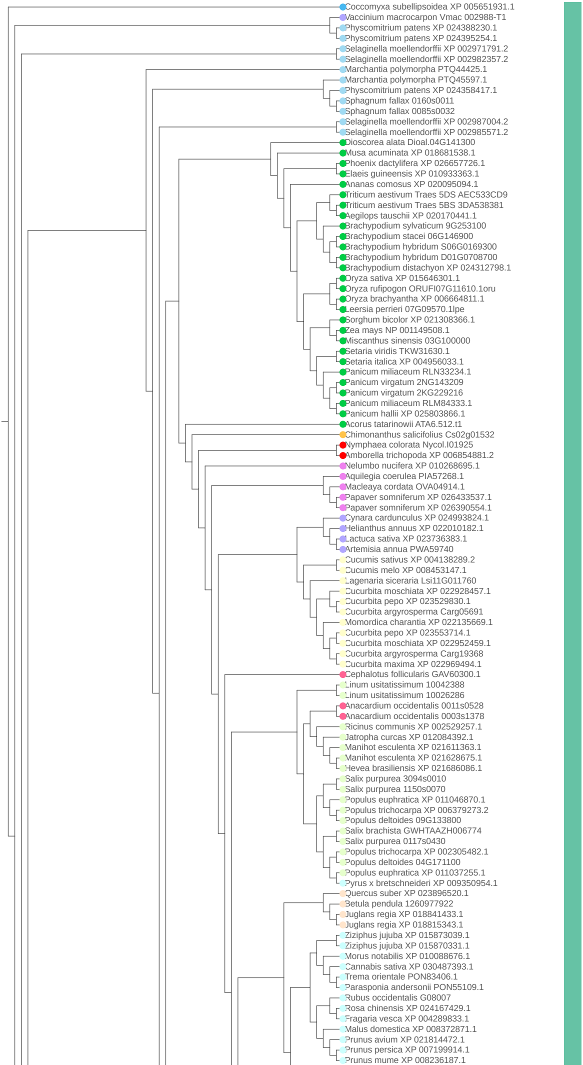
Coccomyxa subellipsoidea XP 005651931.1
Vaccinium macrocarpon Vmac 002988-T1
Physcomitrium patens XP 024388230.1
Physcomitrium patens XP 024395254.1
Selaginella moellendorffii XP 002971791.2
Selaginella moellendorffii XP 002982357.2
Marchantia polymorpha PTQ44425.1
Marchantia polymorpha PTQ45597.1
Physcomitrium patens XP 024358417.1
Sphagnum fallax 0160s0011
Sphagnum fallax 0085s0032
Selaginella moellendorffii XP 002987004.2
Selaginella moellendorffii XP 002985571.2
Dioscorea alata Dioal.04G141300
Musa acuminata XP 018681538.1
Phoenix dactylifera XP 026657726.1
Elaeis guineensis XP 010933363.1
Ananas comosus XP 020095094.1
Triticum aestivum Traes 5DS AEC533CD9
Triticum aestivum Traes 5BS 3DA538381
Aegilops tauschii XP 020170441.1
Brachypodium sylvaticum 9G253100
Brachypodium stacei 06G146900
Brachypodium hybridum S06G0169300
Brachypodium hybridum D01G0708700
Brachypodium distachyon XP 024312798.1
Oryza sativa XP 015646301.1
Oryza rufipogon ORUFI07G11610.1oru
Oryza brachyantha XP 006664811.1
Leersia perrieri 07G09570.1lpe
Sorghum bicolor XP 021308366.1
Zea mays NP 001149508.1
Miscanthus sinensis 03G100000
Setaria viridis TKW31630.1
Setaria italica XP 004956033.1
Panicum miliaceum RLN33234.1
Panicum virgatum 2NG143209
Panicum virgatum 2KG229216
Panicum miliaceum RLM84333.1
Panicum hallii XP 025803866.1
Acorus tatarinowii ATA6.512.t1
Chimonanthus salicifolius Cs02g01532
Nymphaea colorata Nycol.I01925
Amborella trichopoda XP 006854881.2
Nelumbo nucifera XP 010268695.1
Aquilegia coerulea PIA57268.1
Macleaya cordata OVA04914.1
Papaver somniferum XP 026433537.1
Papaver somniferum XP 026390554.1
Cynara cardunculus XP 024993824.1
Helianthus annuus XP 022010182.1
Lactuca sativa XP 023736383.1
Artemisia annua PWA59740
Cucumis sativus XP 004138289.2
Cucumis melo XP 008453147.1
Lagenaria siceraria Lsi11G011760
Cucurbita moschiata XP 022928457.1
Cucurbita pepo XP 023529830.1
Cucurbita argyrosperma Carg05691
Momordica charantia XP 022135669.1
Cucurbita pepo XP 023553714.1
Cucurbita moschiata XP 022952459.1
Cucurbita argyrosperma Carg19368
Cucurbita maxima XP 022969494.1
Cephalotus follicularis GAV60300.1
Linum usitatissimum 10042388
Linum usitatissimum 10026286
Anacardium occidentalis 0011s0528
Anacardium occidentalis 0003s1378
Ricinus communis XP 002529257.1
Jatropha curcas XP 012084392.1
Manihot esculenta XP 021611363.1
Manihot esculenta XP 021628675.1
Hevea brasiliensis XP 021686086.1
Salix purpurea 3094s0010
Salix purpurea 1150s0070
Populus euphratica XP 011046870.1
Populus trichocarpa XP 006379273.2
Populus deltoides 09G133800
Salix brachista GWHTAAZH006774
Salix purpurea 0117s0430
Populus trichocarpa XP 002305482.1
Populus deltoides 04G171100
Populus euphratica XP 011037255.1
Pyrus x bretschneideri XP 009350954.1
Quercus suber XP 023896520.1
Betula pendula 1260977922
Juglans regia XP 018841433.1
Juglans regia XP 018815343.1
Ziziphus jujuba XP 015873039.1
Ziziphus jujuba XP 015870331.1
Morus notabilis XP 010088676.1
Cannabis sativa XP 030487393.1
Trema orientale PON83406.1
Parasponia andersonii PON55109.1
Rubus occidentalis G08007
Rosa chinensis XP 024167429.1
Fragaria vesca XP 004289833.1
Malus domestica XP 008372871.1
Prunus avium XP 021814472.1
Prunus persica XP 007199914.1
Prunus mume XP 008236187.1

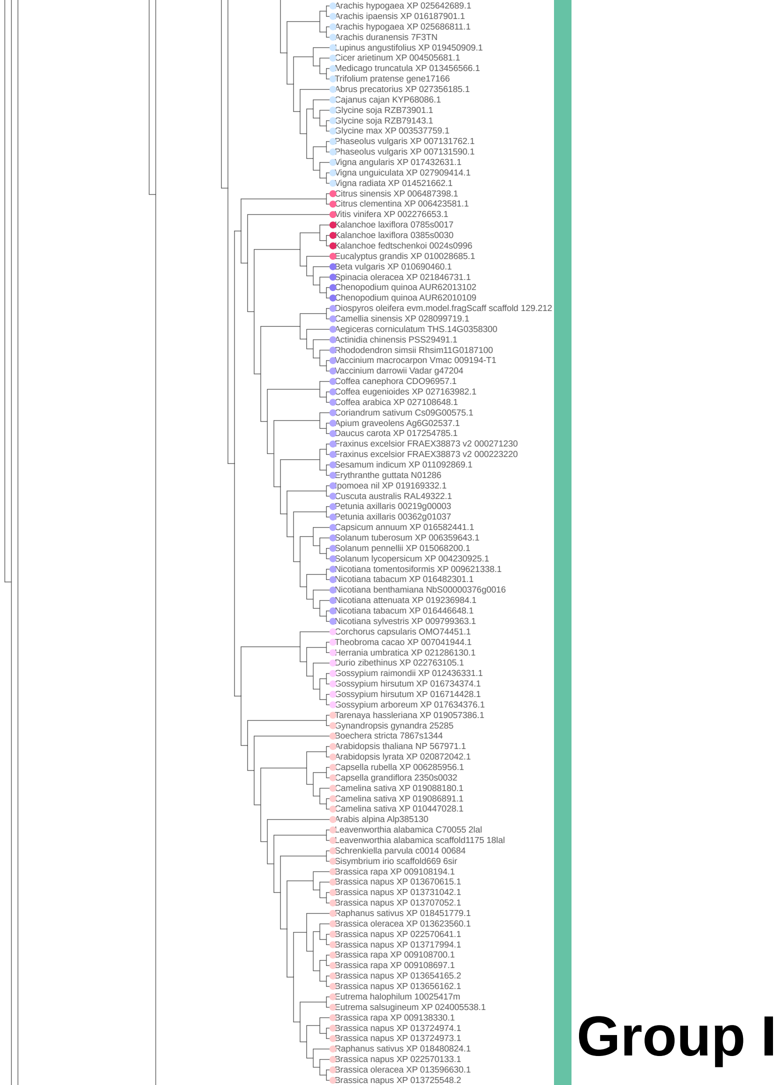

Arachis hypogaea XP 025642689.1
Arachis ipaensis XP 016187901.1
Arachis hypogaea XP 025686811.1
Arachis duranensis 7F3TN
Lupinus angustifolius XP 019450909.1
Cicer arietinum XP 004505681.1
Medicago truncatula XP 013456566.1
Trifolium pratense gene17166
Abrus precatorius XP 027356185.1
Cajanus cajan KYP68086.1
Glycine soja RZB73901.1
Glycine soja RZB79143.1
Glycine max XP 003537759.1
Phaseolus vulgaris XP 007131762.1
Phaseolus vulgaris XP 007131590.1
Vigna angularis XP 017432631.1
Vigna unguiculata XP 027909414.1
Vigna radiata XP 014521662.1
Citrus sinensis XP 006487398.1
Citrus clementina XP 006423581.1
Vitis vinifera XP 002276653.1
Kalanchoe laxiflora 0785s0017
Kalanchoe laxiflora 0385s0030
Kalanchoe fedtschenkoi 0024s0996
Eucalyptus grandis XP 010028685.1
Beta vulgaris XP 010690460.1
Spinacia oleracea XP 021846731.1
Chenopodium quinoa AUR62013102
Chenopodium quinoa AUR62010109
Diospyros oleifera evm.model.fragScaff scaffold 129.212
Camellia sinensis XP 028099719.1
Aegiceras corniculatum THS.14G0358300
Actinidia chinensis PSS29491.1
Rhododendron simsii Rhsim11G0187100
Vaccinium macrocarpon Vmac 009194-T1
Vaccinium darrowii Vadar g47204
Coffea canephora CDO96957.1
Coffea eugenioides XP 027163982.1
Coffea arabica XP 027108648.1
Coriandrum sativum Cs09G00575.1
Apium graveolens Ag6G02537.1
Daucus carota XP 017254785.1
Fraxinus excelsior FRAEX38873 v2 000271230
Fraxinus excelsior FRAEX38873 v2 000223220
Sesamum indicum XP 011092869.1
Erythranthe guttata N01286
Ipomoea nil XP 019169332.1
Cuscuta australis RAL49322.1
Petunia axillaris 00219g00003
Petunia axillaris 00362g01037
Capsicum annuum XP 016582441.1
Solanum tuberosum XP 006359643.1
Solanum pennellii XP 015068200.1
Solanum lycopersicum XP 004230925.1
Nicotiana tomentosiformis XP 009621338.1
Nicotiana tabacum XP 016482301.1
Nicotiana benthamiana NbS00000376g0016
Nicotiana attenuata XP 019236984.1
Nicotiana tabacum XP 016446648.1
Nicotiana sylvestris XP 009799363.1
Corchorus capsularis OMO74451.1
Theobroma cacao XP 007041944.1
Herrania umbratica XP 021286130.1
Durio zibethinus XP 022763105.1
Gossypium raimondii XP 012436331.1
Gossypium hirsutum XP 016734374.1
Gossypium hirsutum XP 016714428.1
Gossypium arboreum XP 017634376.1
Tarenaya hassleriana XP 019057386.1
Gynandropsis gynandra 25285
Boechera stricta 7867s1344
Arabidopsis thaliana NP 567971.1
Arabidopsis lyrata XP 020872042.1
Capsella rubella XP 006285956.1
Capsella grandiflora 2350s0032
Camelina sativa XP 019088180.1
Camelina sativa XP 019086891.1
Camelina sativa XP 010447028.1
Arabis alpina Alp385130
Leavenworthia alabamica C70055 2lal
Leavenworthia alabamica scaffold1175 18lal
Schrenkiella parvula c0014 00684
Sisymbrium irio scaffold669 6sir
Brassica rapa XP 009108194.1
Brassica napus XP 013670615.1
Brassica napus XP 013731042.1
Brassica napus XP 013707052.1
Raphanus sativus XP 018451779.1
Brassica oleracea XP 013623560.1
Brassica napus XP 022570641.1
Brassica napus XP 013717994.1
Brassica rapa XP 009108700.1
Brassica rapa XP 009108697.1
Brassica napus XP 013654165.2
Brassica napus XP 013656162.1
Eutrema halophilum 10025417m
Eutrema salsugineum XP 024005538.1
Brassica rapa XP 009138330.1
Brassica napus XP 013724974.1
Brassica napus XP 013724973.1
Raphanus sativus XP 018480824.1
Brassica napus XP 022570133.1
Brassica oleracea XP 013596630.1
Brassica napus XP 013725548.2
Group I

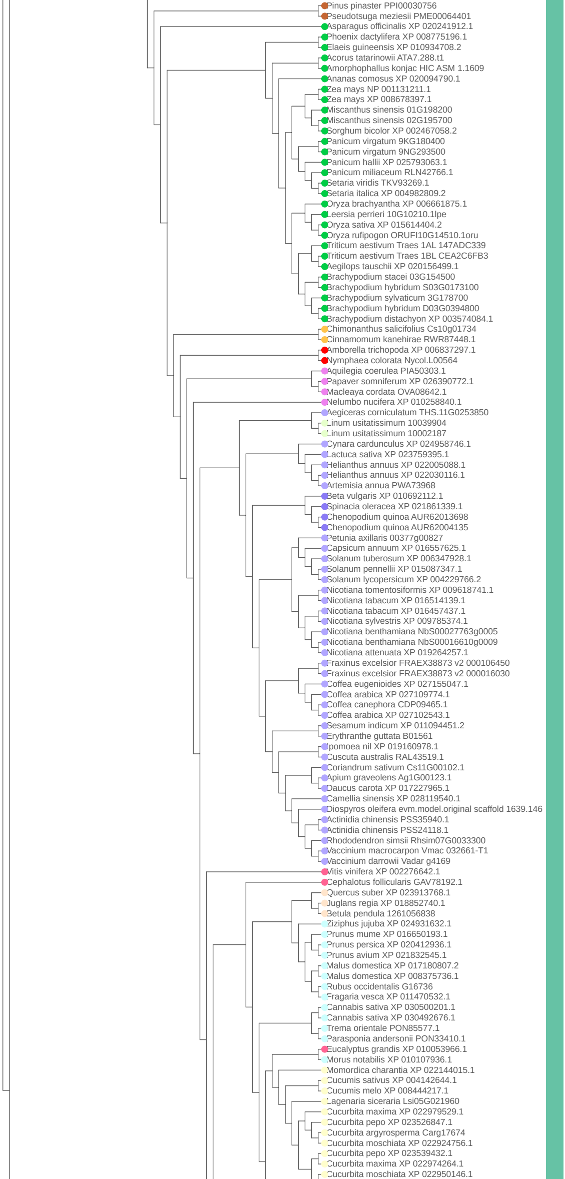
Pinus pinaster PPI00030756
Pseudotsuga meziesii PME00064401
Asparagus officinalis XP 020241912.1
Phoenix dactylifera XP 008775196.1
Elaeis guineensis XP 010934708.2
Acorus tatarinowii ATA7.288.t1
Amorphophallus konjac HIC ASM 1.1609
Ananas comosus XP 020094790.1
Zea mays NP 001131211.1
Zea mays XP 008678397.1
Miscanthus sinensis 01G198200
Miscanthus sinensis 02G195700
Sorghum bicolor XP 002467058.2
Panicum virgatum 9KG180400
Panicum virgatum 9NG293500
Panicum hallii XP 025793063.1
Panicum miliaceum RLN42766.1
Setaria viridis TKV93269.1
Setaria italica XP 004982809.2
Oryza brachyantha XP 006661875.1
Leersia perrieri 10G10210.1lpe
Oryza sativa XP 015614404.2
Oryza rufipogon ORUFI10G14510.1oru
Triticum aestivum Traes 1AL 147ADC339
Triticum aestivum Traes 1BL CEA2C6FB3
Aegilops tauschii XP 020156499.1
Brachypodium stacei 03G154500
Brachypodium hybridum S03G0173100
Brachypodium sylvaticum 3G178700
Brachypodium hybridum D03G0394800
Brachypodium distachyon XP 003574084.1
Chimonanthus salicifolius Cs10g01734
Cinnamomum kanehirae RWR87448.1
Amborella trichopoda XP 006837297.1
Nymphaea colorata Nycol.L00564
Aquilegia coerulea PIA50303.1
Papaver somniferum XP 026390772.1
Macleaya cordata OVA08642.1
Nelumbo nucifera XP 010258840.1
Aegiceras corniculatum THS.11G0253850
Linum usitatissimum 10039904
Linum usitatissimum 10002187
Cynara cardunculus XP 024958746.1
Lactuca sativa XP 023759395.1
Helianthus annuus XP 022005088.1
Helianthus annuus XP 022030116.1
Artemisia annua PWA73968
Beta vulgaris XP 010692112.1
Spinacia oleracea XP 021861339.1
Chenopodium quinoa AUR62013698
Chenopodium quinoa AUR62004135
Petunia axillaris 00377g00827
Capsicum annuum XP 016557625.1
Solanum tuberosum XP 006347928.1
Solanum pennellii XP 015087347.1
Solanum lycopersicum XP 004229766.2
Nicotiana tomentosiformis XP 009618741.1
Nicotiana tabacum XP 016514139.1
Nicotiana tabacum XP 016457437.1
Nicotiana sylvestris XP 009785374.1
Nicotiana benthamiana NbS00027763g0005
Nicotiana benthamiana NbS00016610g0009
Nicotiana attenuata XP 019264257.1
Fraxinus excelsior FRAEX38873 v2 000106450
Fraxinus excelsior FRAEX38873 v2 000016030
Coffea eugenioides XP 027155047.1
Coffea arabica XP 027109774.1
Coffea canephora CDP09465.1
Coffea arabica XP 027102543.1
Sesamum indicum XP 011094451.2
Erythranthe guttata B01561
Ipomoea nil XP 019160978.1
Cuscuta australis RAL43519.1
Coriandrum sativum Cs11G00102.1
Apium graveolens Ag1G00123.1
Daucus carota XP 017227965.1
Camellia sinensis XP 028119540.1
Diospyros oleifera evm.model.original scaffold 1639.146
Actinidia chinensis PSS35940.1
Actinidia chinensis PSS24118.1
Rhododendron simsii Rhsim07G0033300
Vaccinium macrocarpon Vmac 032661-T1
Vaccinium darrowii Vadar g4169
Vitis vinifera XP 002276642.1
Cephalotus follicularis GAV78192.1
Quercus suber XP 023913768.1
Juglans regia XP 018852740.1
Betula pendula 1261056838
Ziziphus jujuba XP 024931632.1
Prunus mume XP 016650193.1
Prunus persica XP 020412936.1
Prunus avium XP 021832545.1
Malus domestica XP 017180807.2
Malus domestica XP 008375736.1
Rubus occidentalis G16736
Fragaria vesca XP 011470532.1
Cannabis sativa XP 030500201.1
Cannabis sativa XP 030492676.1
Trema orientale PON85577.1
Parasponia andersonii PON33410.1
Eucalyptus grandis XP 010053966.1
Morus notabilis XP 010107936.1
Momordica charantia XP 022144015.1
Cucumis sativus XP 004142644.1
Cucumis melo XP 008444217.1
Lagenaria siceraria Lsi05G021960
Cucurbita maxima XP 022979529.1
Cucurbita pepo XP 023526847.1
Cucurbita argyrosperma Carg17674
Cucurbita moschiata XP 022924756.1
Cucurbita pepo XP 023539432.1
Cucurbita maxima XP 022974264.1
Cucurbita moschiata XP 022950146.1

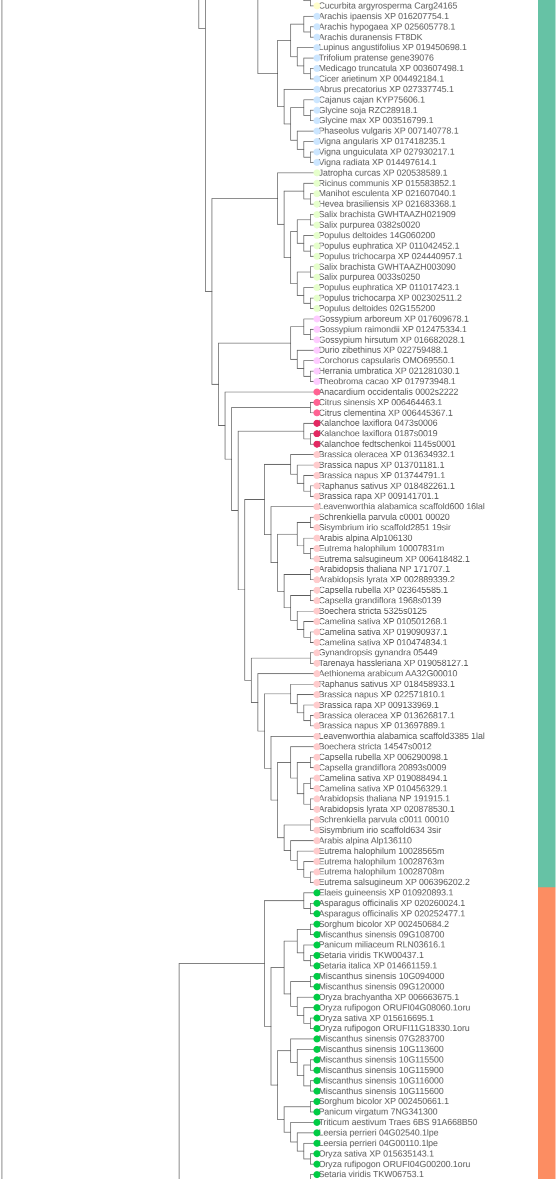

Cucurbita argyrosperma Carg24165
Arachis ipaensis XP 016207754.1
Arachis hypogaea XP 025605778.1
Arachis duranensis FT8DK
Lupinus angustifolius XP 019450698.1
Trifolium pratense gene39076
Medicago truncatula XP 003607498.1
Cicer arietinum XP 004492184.1
Abrus precatorius XP 027337745.1
Cajanus cajan KYP75606.1
Glycine soja RZC28918.1
Glycine max XP 003516799.1
Phaseolus vulgaris XP 007140778.1
Vigna angularis XP 017418235.1
Vigna unguiculata XP 027930217.1
Vigna radiata XP 014497614.1
Jatropha curcas XP 020538589.1
Ricinus communis XP 015583852.1
Manihot esculenta XP 021607040.1
Hevea brasiliensis XP 021683368.1
Salix brachista GWHTAAZH021909
Salix purpurea 0382s0020
Populus deltoides 14G060200
Populus euphratica XP 011042452.1
Populus trichocarpa XP 024440957.1
Salix brachista GWHTAAZH003090
Salix purpurea 0033s0250
Populus euphratica XP 011017423.1
Populus trichocarpa XP 002302511.2
Populus deltoides 02G155200
Gossypium arboreum XP 017609678.1
Gossypium raimondii XP 012475334.1
Gossypium hirsutum XP 016682028.1
Durio zibethinus XP 022759488.1
Corchorus capsularis OMO69550.1
Herrania umbratica XP 021281030.1
Theobroma cacao XP 017973948.1
Anacardium occidentalis 0002s2222
Citrus sinensis XP 006464463.1
Citrus clementina XP 006445367.1
Kalanchoe laxiflora 0473s0006
Kalanchoe laxiflora 0187s0019
Kalanchoe fedtschenkoi 1145s0001
Brassica oleracea XP 013634932.1
Brassica napus XP 013701181.1
Brassica napus XP 013744791.1
Raphanus sativus XP 018482261.1
Brassica rapa XP 009141701.1
Leavenworthia alabamica scaffold600 16lal
Schrenkiella parvula c0001 00020
Sisymbrium irio scaffold2851 19sir
Arabis alpina Alp106130
Eutrema halophilum 10007831m
Eutrema salsugineum XP 006418482.1
Arabidopsis thaliana NP 171707.1
Arabidopsis lyrata XP 002889339.2
Capsella rubella XP 023645585.1
Capsella grandiflora 1968s0139
Boechera stricta 5325s0125
Camelina sativa XP 010501268.1
Camelina sativa XP 019090937.1
Camelina sativa XP 010474834.1
Gynandropsis gynandra 05449
Tarenaya hassleriana XP 019058127.1
Aethionema arabicum AA32G00010
Raphanus sativus XP 018458933.1
Brassica napus XP 022571810.1
Brassica rapa XP 009133969.1
Brassica oleracea XP 013626817.1
Brassica napus XP 013697889.1
Leavenworthia alabamica scaffold3385 1lal
Boechera stricta 14547s0012
Capsella rubella XP 006290098.1
Capsella grandiflora 20893s0009
Camelina sativa XP 019088494.1
Camelina sativa XP 010456329.1
Arabidopsis thaliana NP 191915.1
Arabidopsis lyrata XP 020878530.1
Schrenkiella parvula c0011 00010
Sisymbrium irio scaffold634 3sir
Arabis alpina Alp136110
Eutrema halophilum 10028565m
Eutrema halophilum 10028763m
Eutrema halophilum 10028708m
Eutrema salsugineum XP 006396202.2
Elaeis guineensis XP 010920893.1
Asparagus officinalis XP 020260024.1
Asparagus officinalis XP 020252477.1
Sorghum bicolor XP 002450684.2
Miscanthus sinensis 09G108700
Panicum miliaceum RLN03616.1
Setaria viridis TKW00437.1
Setaria italica XP 014661159.1
Miscanthus sinensis 10G094000
Miscanthus sinensis 09G120000
Oryza brachyantha XP 006663675.1
Oryza rufipogon ORUFI04G08060.1oru
Oryza sativa XP 015616695.1
Oryza rufipogon ORUFI11G18330.1oru
Miscanthus sinensis 07G283700
Miscanthus sinensis 10G113600
Miscanthus sinensis 10G115500
Miscanthus sinensis 10G115900
Miscanthus sinensis 10G116000
Miscanthus sinensis 10G115600
Sorghum bicolor XP 002450661.1
Panicum virgatum 7NG341300
Triticum aestivum Traes 6BS 91A668B50
Leersia perrieri 04G02540.1lpe
Leersia perrieri 04G00110.1lpe
Oryza sativa XP 015635143.1
Oryza rufipogon ORUFI04G00200.1oru
Setaria viridis TKW06753.1

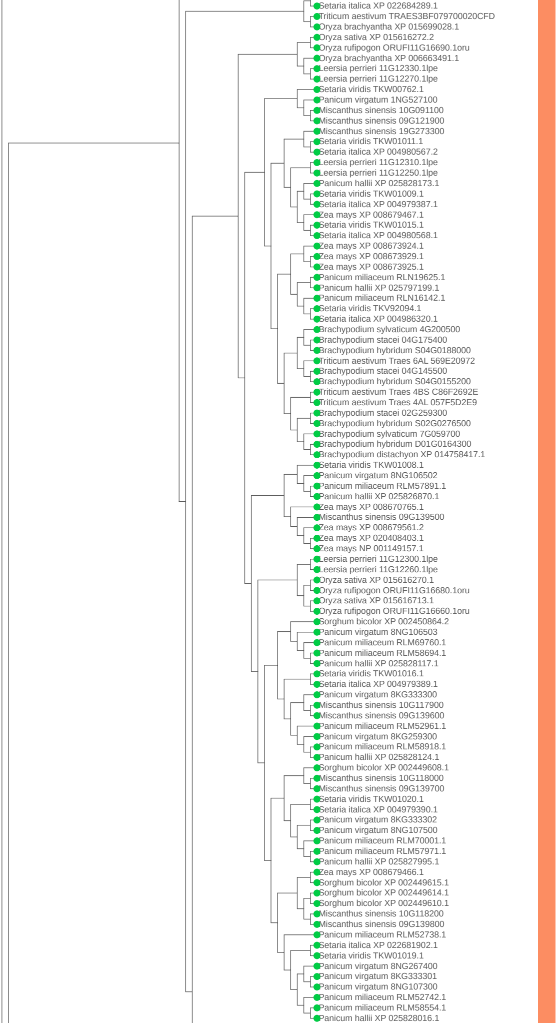
Setaria italica XP 022684289.1
Triticum aestivum TRAES3BF079700020CFD
Oryza brachyantha XP 015699028.1
Oryza sativa XP 015616272.2
Oryza rufipogon ORUFI11G16690.1oru
Oryza brachyantha XP 006663491.1
Leersia perrieri 11G12330.1lpe
Leersia perrieri 11G12270.1lpe
Setaria viridis TKW00762.1
Panicum virgatum 1NG527100
Miscanthus sinensis 10G091100
Miscanthus sinensis 09G121900
Miscanthus sinensis 19G273300
Setaria viridis TKW01011.1
Setaria italica XP 004980567.2
Leersia perrieri 11G12310.1lpe
Leersia perrieri 11G12250.1lpe
Panicum hallii XP 025828173.1
Setaria viridis TKW01009.1
Setaria italica XP 004979387.1
Zea mays XP 008679467.1
Setaria viridis TKW01015.1
Setaria italica XP 004980568.1
Zea mays XP 008673924.1
Zea mays XP 008673929.1
Zea mays XP 008673925.1
Panicum miliaceum RLN19625.1
Panicum hallii XP 025797199.1
Panicum miliaceum RLN16142.1
Setaria viridis TKV92094.1
Setaria italica XP 004986320.1
Brachypodium sylvaticum 4G200500
Brachypodium stacei 04G175400
Brachypodium hybridum S04G0188000
Triticum aestivum Traes 6AL 569E20972
Brachypodium stacei 04G145500
Brachypodium hybridum S04G0155200
Triticum aestivum Traes 4BS C86F2692E
Triticum aestivum Traes 4AL 057F5D2E9
Brachypodium stacei 02G259300
Brachypodium hybridum S02G0276500
Brachypodium sylvaticum 7G059700
Brachypodium hybridum D01G0164300
Brachypodium distachyon XP 014758417.1
Setaria viridis TKW01008.1
Panicum virgatum 8NG106502
Panicum miliaceum RLM57891.1
Panicum hallii XP 025826870.1
Zea mays XP 008670765.1
Miscanthus sinensis 09G139500
Zea mays XP 008679561.2
Zea mays XP 020408403.1
Zea mays NP 001149157.1
Leersia perrieri 11G12300.1lpe
Leersia perrieri 11G12260.1lpe
Oryza sativa XP 015616270.1
Oryza rufipogon ORUFI11G16680.1oru
Oryza sativa XP 015616713.1
Oryza rufipogon ORUFI11G16660.1oru
Sorghum bicolor XP 002450864.2
Panicum virgatum 8NG106503
Panicum miliaceum RLM69760.1
Panicum miliaceum RLM58694.1
Panicum hallii XP 025828117.1
Setaria viridis TKW01016.1
Setaria italica XP 004979389.1
Panicum virgatum 8KG333300
Miscanthus sinensis 10G117900
Miscanthus sinensis 09G139600
Panicum miliaceum RLM52961.1
Panicum virgatum 8KG259300
Panicum miliaceum RLM58918.1
Panicum hallii XP 025828124.1
Sorghum bicolor XP 002449608.1
Miscanthus sinensis 10G118000
Miscanthus sinensis 09G139700
Setaria viridis TKW01020.1
Setaria italica XP 004979390.1
Panicum virgatum 8KG333302
Panicum virgatum 8NG107500
Panicum miliaceum RLM70001.1
Panicum miliaceum RLM57971.1
Panicum hallii XP 025827995.1
Zea mays XP 008679466.1
Sorghum bicolor XP 002449615.1
Sorghum bicolor XP 002449614.1
Sorghum bicolor XP 002449610.1
Miscanthus sinensis 10G118200
Miscanthus sinensis 09G139800
Panicum miliaceum RLM52738.1
Setaria italica XP 022681902.1
Setaria viridis TKW01019.1
Panicum virgatum 8NG267400
Panicum virgatum 8KG333301
Panicum virgatum 8NG107300
Panicum miliaceum RLM52742.1
Panicum miliaceum RLM58554.1
Panicum hallii XP 025828016.1

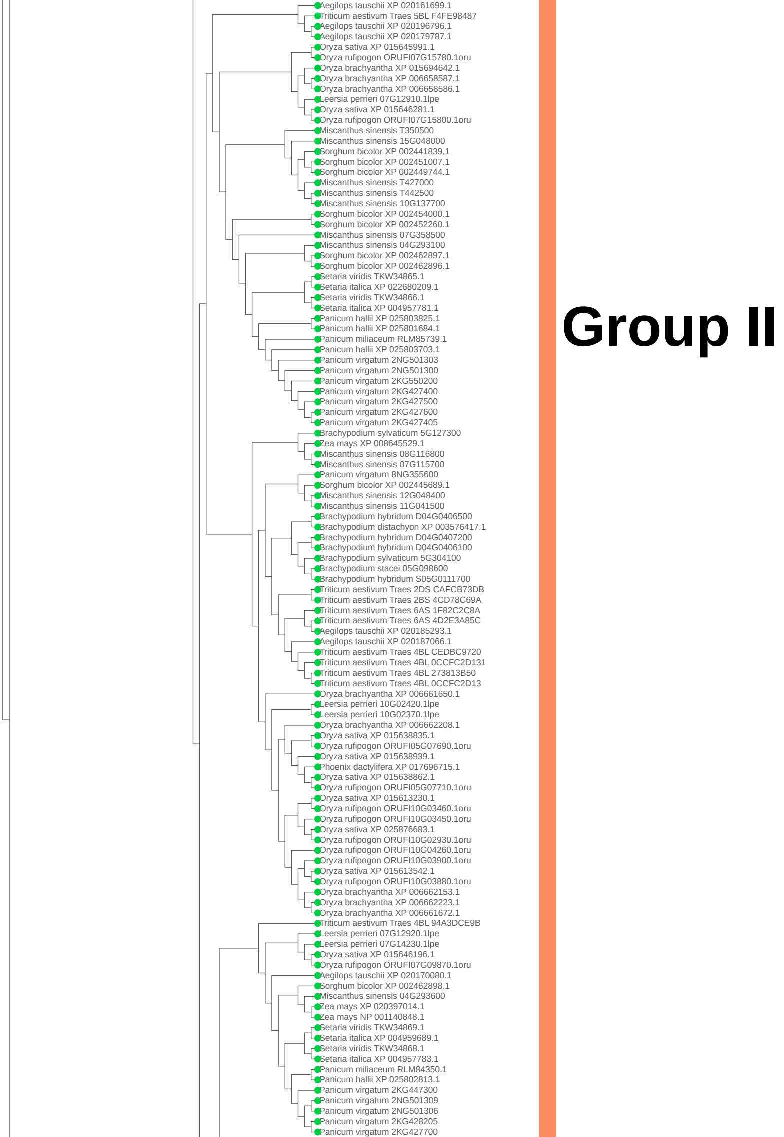
Aegilops tauschii XP 020161699.1
Triticum aestivum Traes 5BL F4FE98487
Aegilops tauschii XP 020196796.1
Aegilops tauschii XP 020179787.1
Oryza sativa XP 015645991.1
Oryza rufipogon ORUFI07G15780.1oru
Oryza brachyantha XP 015694642.1
Oryza brachyantha XP 006658587.1
Oryza brachyantha XP 006658586.1
Leersia perrieri 07G12910.1lpe
Oryza sativa XP 015646281.1
Oryza rufipogon ORUFI07G15800.1oru
Miscanthus sinensis T350500
Miscanthus sinensis 15G048000
Sorghum bicolor XP 002441839.1
Sorghum bicolor XP 002451007.1
Sorghum bicolor XP 002449744.1
Miscanthus sinensis T427000
Miscanthus sinensis T442500
Miscanthus sinensis 10G137700
Sorghum bicolor XP 002454000.1
Sorghum bicolor XP 002452260.1
Miscanthus sinensis 07G358500
Miscanthus sinensis 04G293100
Sorghum bicolor XP 002462897.1
Sorghum bicolor XP 002462896.1
Setaria viridis TKW34865.1
Setaria italica XP 022680209.1
Setaria viridis TKW34866.1
Setaria italica XP 004957781.1
Panicum hallii XP 025803825.1
Panicum hallii XP 025801684.1
Panicum miliaceum RLM85739.1
Panicum hallii XP 025803703.1
Panicum virgatum 2NG501303
Panicum virgatum 2NG501300
Panicum virgatum 2KG550200
Panicum virgatum 2KG427400
Panicum virgatum 2KG427500
Panicum virgatum 2KG427600
Panicum virgatum 2KG427405
Brachypodium sylvaticum 5G127300
Zea mays XP 008645529.1
Miscanthus sinensis 08G116800
Miscanthus sinensis 07G115700
Panicum virgatum 8NG355600
Sorghum bicolor XP 002445689.1
Miscanthus sinensis 12G048400
Miscanthus sinensis 11G041500
Brachypodium hybridum D04G0406500
Brachypodium distachyon XP 003576417.1
Brachypodium hybridum D04G0407200
Brachypodium hybridum D04G0406100
Brachypodium sylvaticum 5G304100
Brachypodium stacei 05G098600
Brachypodium hybridum S05G0111700
Triticum aestivum Traes 2DS CAFCB73DB
Triticum aestivum Traes 2BS 4CD78C69A
Triticum aestivum Traes 6AS 1F82C2C8A
Triticum aestivum Traes 6AS 4D2E3A85C
Aegilops tauschii XP 020185293.1
Aegilops tauschii XP 020187066.1
Triticum aestivum Traes 4BL CEDBC9720
Triticum aestivum Traes 4BL 0CCFC2D131
Triticum aestivum Traes 4BL 273813B50
Triticum aestivum Traes 4BL 0CCFC2D13
Oryza brachyantha XP 006661650.1
Leersia perrieri 10G02420.1lpe
Leersia perrieri 10G02370.1lpe
Oryza brachyantha XP 006662208.1
Oryza sativa XP 015638835.1
Oryza rufipogon ORUFI05G07690.1oru
Oryza sativa XP 015638939.1
Phoenix dactylifera XP 017696715.1
Oryza sativa XP 015638862.1
Oryza rufipogon ORUFI05G07710.1oru
Oryza sativa XP 015613230.1
Oryza rufipogon ORUFI10G03460.1oru
Oryza rufipogon ORUFI10G03450.1oru
Oryza sativa XP 025876683.1
Oryza rufipogon ORUFI10G02930.1oru
Oryza rufipogon ORUFI10G04260.1oru
Oryza rufipogon ORUFI10G03900.1oru
Oryza sativa XP 015613542.1
Oryza rufipogon ORUFI10G03880.1oru
Oryza brachyantha XP 006662153.1
Oryza brachyantha XP 006662223.1
Oryza brachyantha XP 006661672.1
Triticum aestivum Traes 4BL 94A3DCE9B
Leersia perrieri 07G12920.1lpe
Leersia perrieri 07G14230.1lpe
Oryza sativa XP 015646196.1
Oryza rufipogon ORUFI07G09870.1oru
Aegilops tauschii XP 020170080.1
Sorghum bicolor XP 002462898.1
Miscanthus sinensis 04G293600
Zea mays XP 020397014.1
Zea mays NP 001140848.1
Setaria viridis TKW34869.1
Setaria italica XP 004959689.1
Setaria viridis TKW34868.1
Setaria italica XP 004957783.1
Panicum miliaceum RLM84350.1
Panicum hallii XP 025802813.1
Panicum virgatum 2KG447300
Panicum virgatum 2NG501309
Panicum virgatum 2NG501306
Panicum virgatum 2KG428205
Panicum virgatum 2KG427700
Group II

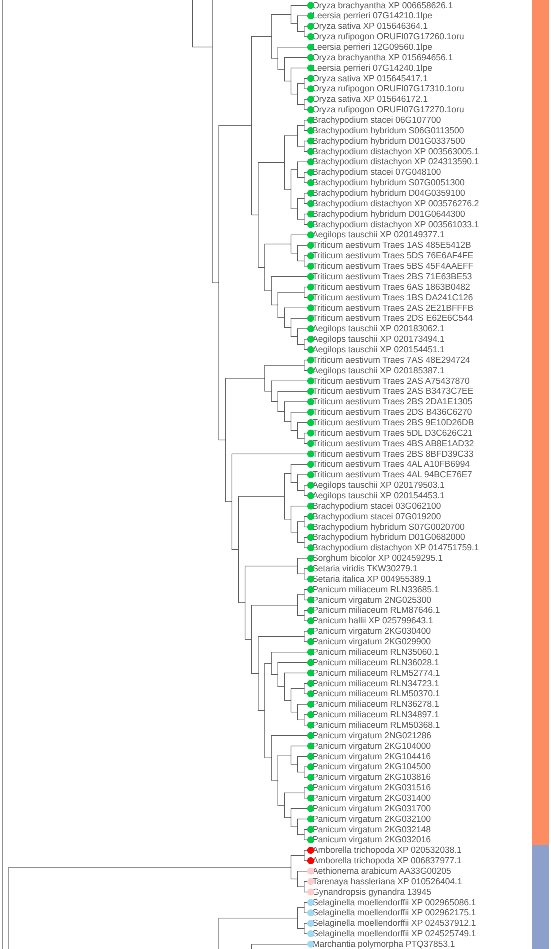

Oryza brachyantha XP 006658626.1
Leersia perrieri 07G14210.1lpe
Oryza sativa XP 015646364.1
Oryza rufipogon ORUFI07G17260.1oru
Leersia perrieri 12G09560.1lpe
Oryza brachyantha XP 015694656.1
Leersia perrieri 07G14240.1lpe
Oryza sativa XP 015645417.1
Oryza rufipogon ORUFI07G17310.1oru
Oryza sativa XP 015646172.1
Oryza rufipogon ORUFI07G17270.1oru
Brachypodium stacei 06G107700
Brachypodium hybridum S06G0113500
Brachypodium hybridum D01G0337500
Brachypodium distachyon XP 003563005.1
Brachypodium distachyon XP 024313590.1
Brachypodium stacei 07G048100
Brachypodium hybridum S07G0051300
Brachypodium hybridum D04G0359100
Brachypodium distachyon XP 003576276.2
Brachypodium hybridum D01G0644300
Brachypodium distachyon XP 003561033.1
Aegilops tauschii XP 020149377.1
Triticum aestivum Traes 1AS 485E5412B
Triticum aestivum Traes 5DS 76E6AF4FE
Triticum aestivum Traes 5BS 45F4AAEFF
Triticum aestivum Traes 2BS 71E63BE53
Triticum aestivum Traes 6AS 1863B0482
Triticum aestivum Traes 1BS DA241C126
Triticum aestivum Traes 2AS 2E21BFFFB
Triticum aestivum Traes 2DS E62E6C544
Aegilops tauschii XP 020183062.1
Aegilops tauschii XP 020173494.1
Aegilops tauschii XP 020154451.1
Triticum aestivum Traes 7AS 48E294724
Aegilops tauschii XP 020185387.1
Triticum aestivum Traes 2AS A75437870
Triticum aestivum Traes 2AS B3473C7EE
Triticum aestivum Traes 2BS 2DA1E1305
Triticum aestivum Traes 2DS B436C6270
Triticum aestivum Traes 2BS 9E10D26DB
Triticum aestivum Traes 5DL D3C626C21
Triticum aestivum Traes 4BS AB8E1AD32
Triticum aestivum Traes 2BS 8BFD39C33
Triticum aestivum Traes 4AL A10FB6994
Triticum aestivum Traes 4AL 94BCE76E7
Aegilops tauschii XP 020179503.1
Aegilops tauschii XP 020154453.1
Brachypodium stacei 03G062100
Brachypodium stacei 07G019200
Brachypodium hybridum S07G0020700
Brachypodium hybridum D01G0682000
Brachypodium distachyon XP 014751759.1
Sorghum bicolor XP 002459295.1
Setaria viridis TKW30279.1
Setaria italica XP 004955389.1
Panicum miliaceum RLN33685.1
Panicum virgatum 2NG025300
Panicum miliaceum RLM87646.1
Panicum hallii XP 025799643.1
Panicum virgatum 2KG030400
Panicum virgatum 2KG029900
Panicum miliaceum RLN35060.1
Panicum miliaceum RLN36028.1
Panicum miliaceum RLM52774.1
Panicum miliaceum RLN34723.1
Panicum miliaceum RLM50370.1
Panicum miliaceum RLN36278.1
Panicum miliaceum RLN34897.1
Panicum miliaceum RLM50368.1
Panicum virgatum 2NG021286
Panicum virgatum 2KG104000
Panicum virgatum 2KG104416
Panicum virgatum 2KG104500
Panicum virgatum 2KG103816
Panicum virgatum 2KG031516
Panicum virgatum 2KG031400
Panicum virgatum 2KG031700
Panicum virgatum 2KG032100
Panicum virgatum 2KG032148
Panicum virgatum 2KG032016
Amborella trichopoda XP 020532038.1
Amborella trichopoda XP 006837977.1
Aethionema arabicum AA33G00205
Tarenaya hassleriana XP 010526404.1
Gynandropsis gynandra 13945
Selaginella moellendorffii XP 002965086.1
Selaginella moellendorffii XP 002962175.1
Selaginella moellendorffii XP 024537912.1
Selaginella moellendorffii XP 024525749.1
Marchantia polymorpha PTQ37853.1

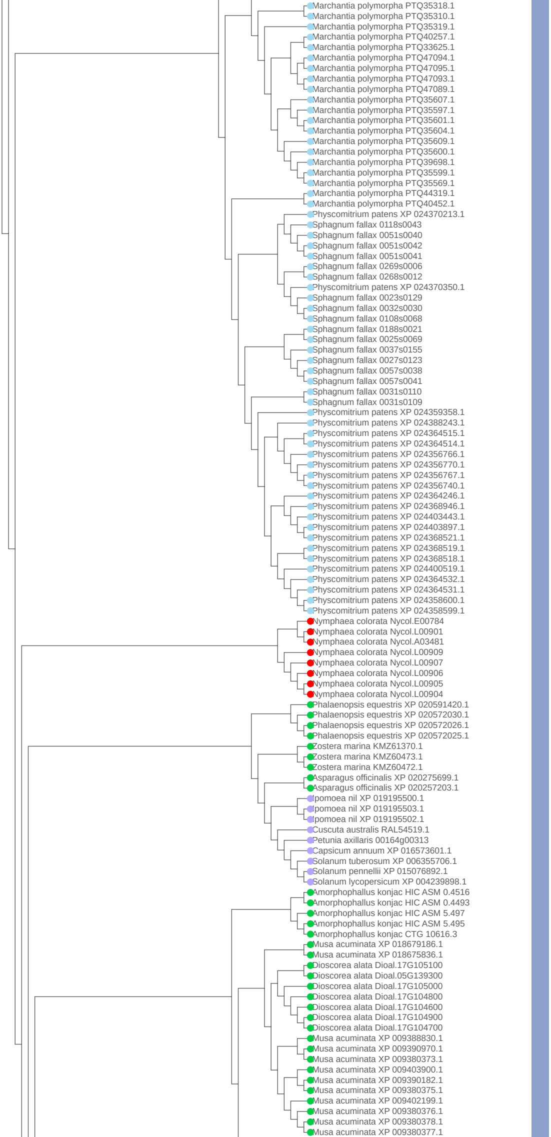
Marchantia polymorpha PTQ35318.1
Marchantia polymorpha PTQ35310.1
Marchantia polymorpha PTQ35319.1
Marchantia polymorpha PTQ40257.1
Marchantia polymorpha PTQ33625.1
Marchantia polymorpha PTQ47094.1
Marchantia polymorpha PTQ47095.1
Marchantia polymorpha PTQ47093.1
Marchantia polymorpha PTQ47089.1
Marchantia polymorpha PTQ35607.1
Marchantia polymorpha PTQ35597.1
Marchantia polymorpha PTQ35601.1
Marchantia polymorpha PTQ35604.1
Marchantia polymorpha PTQ35609.1
Marchantia polymorpha PTQ35600.1
Marchantia polymorpha PTQ39698.1
Marchantia polymorpha PTQ35599.1
Marchantia polymorpha PTQ35569.1
Marchantia polymorpha PTQ44319.1
Marchantia polymorpha PTQ40452.1
Physcomitrium patens XP 024370213.1
Sphagnum fallax 0118s0043
Sphagnum fallax 0051s0040
Sphagnum fallax 0051s0042
Sphagnum fallax 0051s0041
Sphagnum fallax 0269s0006
Sphagnum fallax 0268s0012
Physcomitrium patens XP 024370350.1
Sphagnum fallax 0023s0129
Sphagnum fallax 0032s0030
Sphagnum fallax 0108s0068
Sphagnum fallax 0188s0021
Sphagnum fallax 0025s0069
Sphagnum fallax 0037s0155
Sphagnum fallax 0027s0123
Sphagnum fallax 0057s0038
Sphagnum fallax 0057s0041
Sphagnum fallax 0031s0110
Sphagnum fallax 0031s0109
Physcomitrium patens XP 024359358.1
Physcomitrium patens XP 024388243.1
Physcomitrium patens XP 024364515.1
Physcomitrium patens XP 024364514.1
Physcomitrium patens XP 024356766.1
Physcomitrium patens XP 024356770.1
Physcomitrium patens XP 024356767.1
Physcomitrium patens XP 024356740.1
Physcomitrium patens XP 024364246.1
Physcomitrium patens XP 024368946.1
Physcomitrium patens XP 024403443.1
Physcomitrium patens XP 024403897.1
Physcomitrium patens XP 024368521.1
Physcomitrium patens XP 024368519.1
Physcomitrium patens XP 024368518.1
Physcomitrium patens XP 024400519.1
Physcomitrium patens XP 024364532.1
Physcomitrium patens XP 024364531.1
Physcomitrium patens XP 024358600.1
Physcomitrium patens XP 024358599.1
Nymphaea colorata Nycol.E00784
Nymphaea colorata Nycol.L00901
Nymphaea colorata Nycol.A03481
Nymphaea colorata Nycol.L00909
Nymphaea colorata Nycol.L00907
Nymphaea colorata Nycol.L00906
Nymphaea colorata Nycol.L00905
Nymphaea colorata Nycol.L00904
Phalaenopsis equestris XP 020591420.1
Phalaenopsis equestris XP 020572030.1
Phalaenopsis equestris XP 020572026.1
Phalaenopsis equestris XP 020572025.1
Zostera marina KMZ61370.1
Zostera marina KMZ60473.1
Zostera marina KMZ60472.1
Asparagus officinalis XP 020275699.1
Asparagus officinalis XP 020257203.1
Ipomoea nil XP 019195500.1
Ipomoea nil XP 019195503.1
Ipomoea nil XP 019195502.1
Cuscuta australis RAL54519.1
Petunia axillaris 00164g00313
Capsicum annuum XP 016573601.1
Solanum tuberosum XP 006355706.1
Solanum pennellii XP 015076892.1
Solanum lycopersicum XP 004239898.1
Amorphophallus konjac HIC ASM 0.4516
Amorphophallus konjac HIC ASM 0.4493
Amorphophallus konjac HIC ASM 5.497
Amorphophallus konjac HIC ASM 5.495
Amorphophallus konjac CTG 10616.3
Musa acuminata XP 018679186.1
Musa acuminata XP 018675836.1
Dioscorea alata Dioal.17G105100
Dioscorea alata Dioal.05G139300
Dioscorea alata Dioal.17G105000
Dioscorea alata Dioal.17G104800
Dioscorea alata Dioal.17G104600
Dioscorea alata Dioal.17G104900
Dioscorea alata Dioal.17G104700
Musa acuminata XP 009388830.1
Musa acuminata XP 009390970.1
Musa acuminata XP 009380373.1
Musa acuminata XP 009403900.1
Musa acuminata XP 009390182.1
Musa acuminata XP 009380375.1
Musa acuminata XP 009402199.1
Musa acuminata XP 009380376.1
Musa acuminata XP 009380378.1
Musa acuminata XP 009380377.1

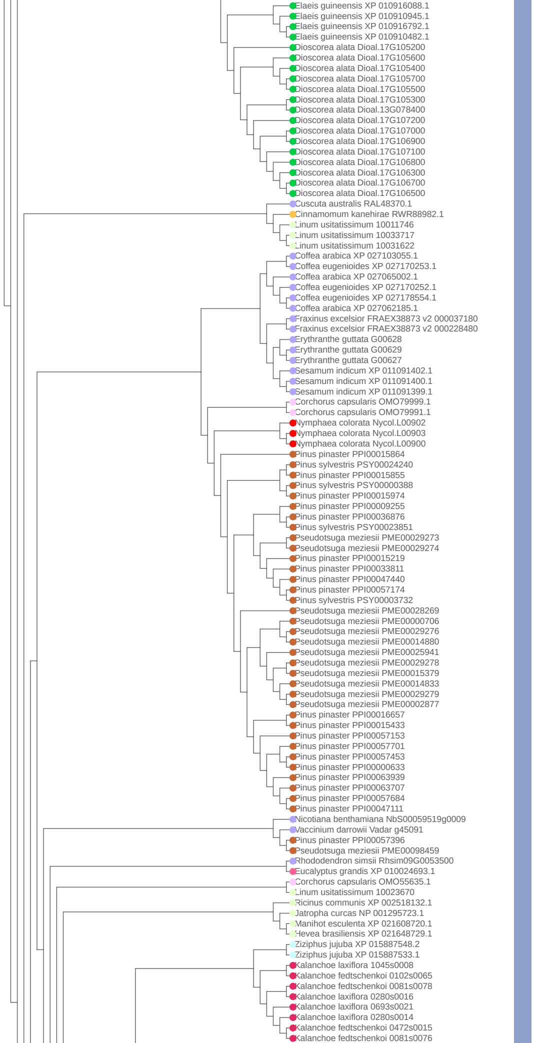

Elaeis guineensis XP 010916088.1
Elaeis guineensis XP 010910945.1
Elaeis guineensis XP 010916792.1
Elaeis guineensis XP 010910482.1
Dioscorea alata Dioal.17G105200
Dioscorea alata Dioal.17G105600
Dioscorea alata Dioal.17G105400
Dioscorea alata Dioal.17G105700
Dioscorea alata Dioal.17G105500
Dioscorea alata Dioal.17G105300
Dioscorea alata Dioal.13G078400
Dioscorea alata Dioal.17G107200
Dioscorea alata Dioal.17G107000
Dioscorea alata Dioal.17G106900
Dioscorea alata Dioal.17G107100
Dioscorea alata Dioal.17G106800
Dioscorea alata Dioal.17G106300
Dioscorea alata Dioal.17G106700
Dioscorea alata Dioal.17G106500
Cuscuta australis RAL48370.1
Cinnamomum kanehirae RWR88982.1
Linum usitatissimum 10011746
Linum usitatissimum 10033717
Linum usitatissimum 10031622
Coffea arabica XP 027103055.1
Coffea eugenioides XP 027170253.1
Coffea arabica XP 027065002.1
Coffea eugenioides XP 027170252.1
Coffea eugenioides XP 027178554.1
Coffea arabica XP 027062185.1
Fraxinus excelsior FRAEX38873 v2 000037180
Fraxinus excelsior FRAEX38873 v2 000228480
Erythranthe guttata G00628
Erythranthe guttata G00629
Erythranthe guttata G00627
Sesamum indicum XP 011091402.1
Sesamum indicum XP 011091400.1
Sesamum indicum XP 011091399.1
Corchorus capsularis OMO79999.1
Corchorus capsularis OMO79991.1
Nymphaea colorata Nycol.L00902
Nymphaea colorata Nycol.L00903
Nymphaea colorata Nycol.L00900
Pinus pinaster PPI00015864
Pinus sylvestris PSY00024240
Pinus pinaster PPI00015855
Pinus sylvestris PSY00000388
Pinus pinaster PPI00015974
Pinus pinaster PPI00009255
Pinus pinaster PPI00036876
Pinus sylvestris PSY00023851
Pseudotsuga meziesii PME00029273
Pseudotsuga meziesii PME00029274
Pinus pinaster PPI00015219
Pinus pinaster PPI00033811
Pinus pinaster PPI00047440
Pinus pinaster PPI00057174
Pinus sylvestris PSY00003732
Pseudotsuga meziesii PME00028269
Pseudotsuga meziesii PME00000706
Pseudotsuga meziesii PME00029276
Pseudotsuga meziesii PME00014880
Pseudotsuga meziesii PME00025941
Pseudotsuga meziesii PME00029278
Pseudotsuga meziesii PME00015379
Pseudotsuga meziesii PME00014833
Pseudotsuga meziesii PME00029279
Pseudotsuga meziesii PME00002877
Pinus pinaster PPI00016657
Pinus pinaster PPI00015433
Pinus pinaster PPI00057153
Pinus pinaster PPI00057701
Pinus pinaster PPI00057453
Pinus pinaster PPI00000633
Pinus pinaster PPI00063939
Pinus pinaster PPI00063707
Pinus pinaster PPI00057684
Pinus pinaster PPI00047111
Nicotiana benthamiana NbS00059519g0009
Vaccinium darrowii Vadar g45091
Pinus pinaster PPI00057396
Pseudotsuga meziesii PME00098459
Rhododendron simsii Rhsim09G0053500
Eucalyptus grandis XP 010024693.1
Corchorus capsularis OMO55635.1
Linum usitatissimum 10023670
Ricinus communis XP 002518132.1
Jatropha curcas NP 001295723.1
Manihot esculenta XP 021608720.1
Hevea brasiliensis XP 021648729.1
Ziziphus jujuba XP 015887548.2
Ziziphus jujuba XP 015887533.1
Kalanchoe laxiflora 1045s0008
Kalanchoe fedtschenkoi 0102s0065
Kalanchoe fedtschenkoi 0081s0078
Kalanchoe laxiflora 0280s0016
Kalanchoe laxiflora 0693s0021
Kalanchoe laxiflora 0280s0014
Kalanchoe fedtschenkoi 0472s0015
Kalanchoe fedtschenkoi 0081s0076

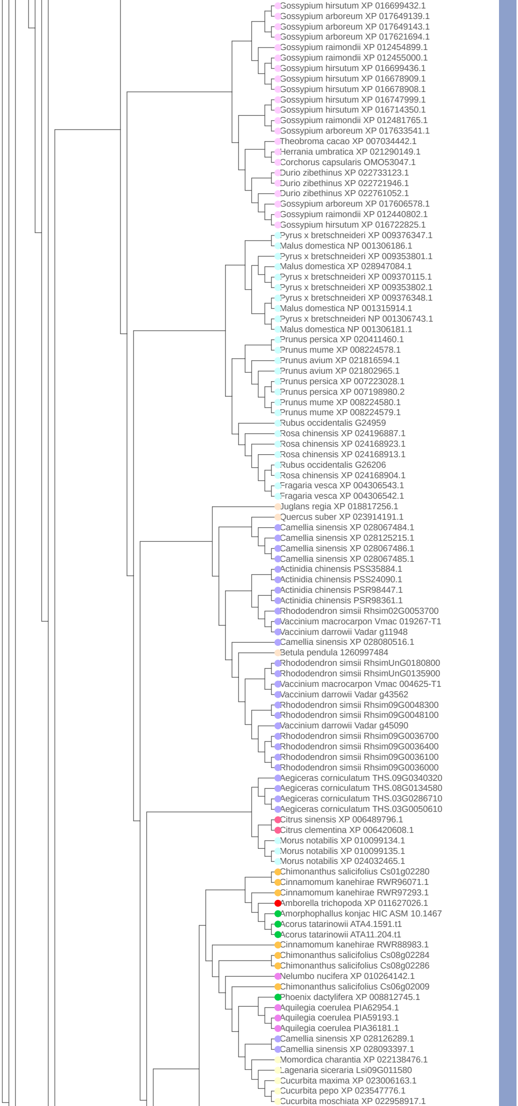
Gossypium hirsutum XP 016699432.1
Gossypium arboreum XP 017649139.1
Gossypium arboreum XP 017649143.1
Gossypium arboreum XP 017621694.1
Gossypium raimondii XP 012454899.1
Gossypium raimondii XP 012455000.1
Gossypium hirsutum XP 016699436.1
Gossypium hirsutum XP 016678909.1
Gossypium hirsutum XP 016678908.1
Gossypium hirsutum XP 016747999.1
Gossypium hirsutum XP 016714350.1
Gossypium raimondii XP 012481765.1
Gossypium arboreum XP 017633541.1
Theobroma cacao XP 007034442.1
Herrania umbratica XP 021290149.1
Corchorus capsularis OMO53047.1
Durio zibethinus XP 022733123.1
Durio zibethinus XP 022721946.1
Durio zibethinus XP 022761052.1
Gossypium arboreum XP 017606578.1
Gossypium raimondii XP 012440802.1
Gossypium hirsutum XP 016722825.1
Pyrus x bretschneideri XP 009376347.1
Malus domestica NP 001306186.1
Pyrus x bretschneideri XP 009353801.1
Malus domestica XP 028947084.1
Pyrus x bretschneideri XP 009370115.1
Pyrus x bretschneideri XP 009353802.1
Pyrus x bretschneideri XP 009376348.1
Malus domestica NP 001315914.1
Pyrus x bretschneideri NP 001306743.1
Malus domestica NP 001306181.1
Prunus persica XP 020411460.1
Prunus mume XP 008224578.1
Prunus avium XP 021816594.1
Prunus avium XP 021802965.1
Prunus persica XP 007223028.1
Prunus persica XP 007198980.2
Prunus mume XP 008224580.1
Prunus mume XP 008224579.1
Rubus occidentalis G24959
Rosa chinensis XP 024196887.1
Rosa chinensis XP 024168923.1
Rosa chinensis XP 024168913.1
Rubus occidentalis G26206
Rosa chinensis XP 024168904.1
Fragaria vesca XP 004306543.1
Fragaria vesca XP 004306542.1
Juglans regia XP 018817256.1
Quercus suber XP 023914191.1
Camellia sinensis XP 028067484.1
Camellia sinensis XP 028125215.1
Camellia sinensis XP 028067486.1
Camellia sinensis XP 028067485.1
Actinidia chinensis PSS35884.1
Actinidia chinensis PSS24090.1
Actinidia chinensis PSR98447.1
Actinidia chinensis PSR98361.1
Rhododendron simsii Rhsim02G0053700
Vaccinium macrocarpon Vmac 019267-T1
Vaccinium darrowii Vadar g11948
Camellia sinensis XP 028080516.1
Betula pendula 1260997484
Rhododendron simsii RhsimUnG0180800
Rhododendron simsii RhsimUnG0135900
Vaccinium macrocarpon Vmac 004625-T1
Vaccinium darrowii Vadar g43562
Rhododendron simsii Rhsim09G0048300
Rhododendron simsii Rhsim09G0048100
Vaccinium darrowii Vadar g45090
Rhododendron simsii Rhsim09G0036700
Rhododendron simsii Rhsim09G0036400
Rhododendron simsii Rhsim09G0036100
Rhododendron simsii Rhsim09G0036000
Aegiceras corniculatum THS.09G0340320
Aegiceras corniculatum THS.08G0134580
Aegiceras corniculatum THS.03G0286710
Aegiceras corniculatum THS.03G0050610
Citrus sinensis XP 006489796.1
Citrus clementina XP 006420608.1
Morus notabilis XP 010099134.1
Morus notabilis XP 010099135.1
Morus notabilis XP 024032465.1
Chimonanthus salicifolius Cs01g02280
Cinnamomum kanehirae RWR96071.1
Cinnamomum kanehirae RWR97293.1
Amborella trichopoda XP 011627026.1
Amorphophallus konjac HIC ASM 10.1467
Acorus tatarinowii ATA4.1591.t1
Acorus tatarinowii ATA11.204.t1
Cinnamomum kanehirae RWR88983.1
Chimonanthus salicifolius Cs08g02284
Chimonanthus salicifolius Cs08g02286
Nelumbo nucifera XP 010264142.1
Chimonanthus salicifolius Cs06g02009
Phoenix dactylifera XP 008812745.1
Aquilegia coerulea PIA62954.1
Aquilegia coerulea PIA59193.1
Aquilegia coerulea PIA36181.1
Camellia sinensis XP 028126289.1
Camellia sinensis XP 028093397.1
Momordica charantia XP 022138476.1
Lagenaria siceraria Lsi09G011580
Cucurbita maxima XP 023006163.1
Cucurbita pepo XP 023547776.1
Cucurbita moschiata XP 022958917.1

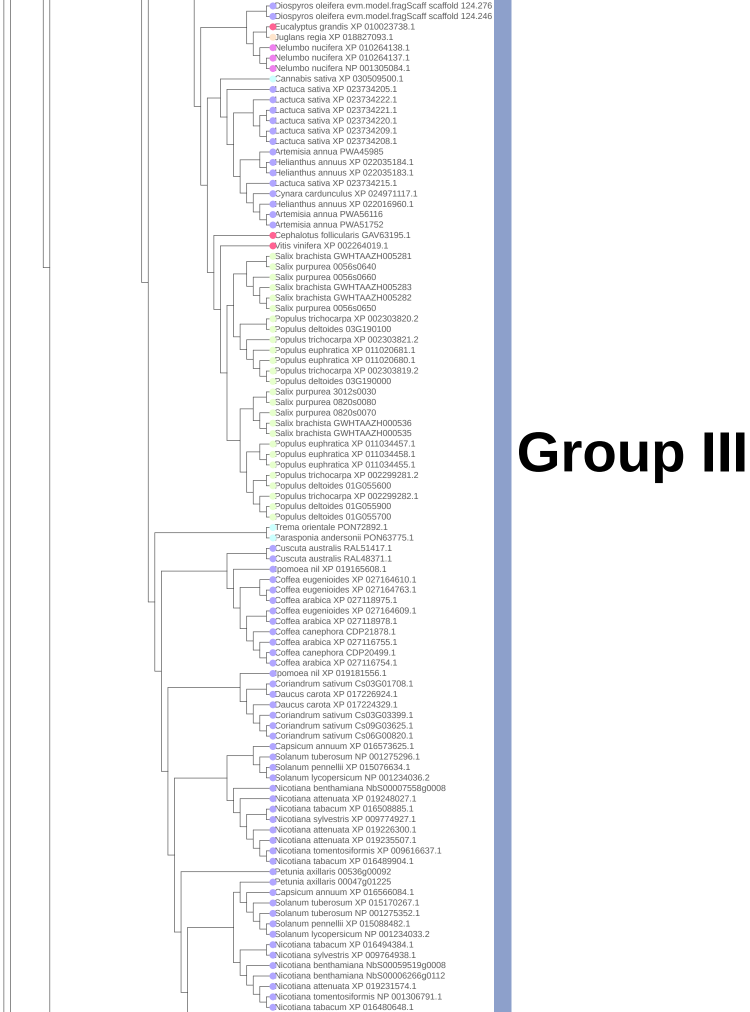
Diospyros oleifera evm.model.fragScaff scaffold 124.276
Diospyros oleifera evm.model.fragScaff scaffold 124.246
Eucalyptus grandis XP 010023738.1
Juglans regia XP 018827093.1
Nelumbo nucifera XP 010264138.1
Nelumbo nucifera XP 010264137.1
Nelumbo nucifera NP 001305084.1
Cannabis sativa XP 030509500.1
Lactuca sativa XP 023734205.1
Lactuca sativa XP 023734222.1
Lactuca sativa XP 023734221.1
Lactuca sativa XP 023734220.1
Lactuca sativa XP 023734209.1
Lactuca sativa XP 023734208.1
Artemisia annua PWA45985
Helianthus annuus XP 022035184.1
Helianthus annuus XP 022035183.1
Lactuca sativa XP 023734215.1
Cynara cardunculus XP 024971117.1
Helianthus annuus XP 022016960.1
Artemisia annua PWA56116
Artemisia annua PWA51752
Cephalotus follicularis GAV63195.1
Vitis vinifera XP 002264019.1
Salix brachista GWHTAAZH005281
Salix purpurea 0056s0640
Salix purpurea 0056s0660
Salix brachista GWHTAAZH005283
Salix brachista GWHTAAZH005282
Salix purpurea 0056s0650
Populus trichocarpa XP 002303820.2
Populus deltoides 03G190100
Populus trichocarpa XP 002303821.2
Populus euphratica XP 011020681.1
Populus euphratica XP 011020680.1
Populus trichocarpa XP 002303819.2
Populus deltoides 03G190000
Salix purpurea 3012s0030
Salix purpurea 0820s0080
Salix purpurea 0820s0070
Salix brachista GWHTAAZH000536
Salix brachista GWHTAAZH000535
Populus euphratica XP 011034457.1
Populus euphratica XP 011034458.1
Populus euphratica XP 011034455.1
Populus trichocarpa XP 002299281.2
Populus deltoides 01G055600
Populus trichocarpa XP 002299282.1
Populus deltoides 01G055900
Populus deltoides 01G055700
Trema orientale PON72892.1
Parasponia andersonii PON63775.1
Cuscuta australis RAL51417.1
Cuscuta australis RAL48371.1
Ipomoea nil XP 019165608.1
Coffea eugenioides XP 027164610.1
Coffea eugenioides XP 027164763.1
Coffea arabica XP 027118975.1
Coffea eugenioides XP 027164609.1
Coffea arabica XP 027118978.1
Coffea canephora CDP21878.1
Coffea arabica XP 027116755.1
Coffea canephora CDP20499.1
Coffea arabica XP 027116754.1
Ipomoea nil XP 019181556.1
Coriandrum sativum Cs03G01708.1
Daucus carota XP 017226924.1
Daucus carota XP 017224329.1
Coriandrum sativum Cs03G03399.1
Coriandrum sativum Cs09G03625.1
Coriandrum sativum Cs06G00820.1
Capsicum annuum XP 016573625.1
Solanum tuberosum NP 001275296.1
Solanum pennellii XP 015076634.1
Solanum lycopersicum NP 001234036.2
Nicotiana benthamiana NbS00007558g0008
Nicotiana attenuata XP 019248027.1
Nicotiana tabacum XP 016508885.1
Nicotiana sylvestris XP 009774927.1
Nicotiana attenuata XP 019226300.1
Nicotiana attenuata XP 019235507.1
Nicotiana tomentosiformis XP 009616637.1
Nicotiana tabacum XP 016489904.1
Petunia axillaris 00536g00092
Petunia axillaris 00047g01225
Capsicum annuum XP 016566084.1
Solanum tuberosum XP 015170267.1
Solanum tuberosum NP 001275352.1
Solanum pennellii XP 015088482.1
Solanum lycopersicum NP 001234033.2
Nicotiana tabacum XP 016494384.1
Nicotiana sylvestris XP 009764938.1
Nicotiana benthamiana NbS00059519g0008
Nicotiana benthamiana NbS00006266g0112
Nicotiana attenuata XP 019231574.1
Nicotiana tomentosiformis NP 001306791.1
Nicotiana tabacum XP 016480648.1
Group III

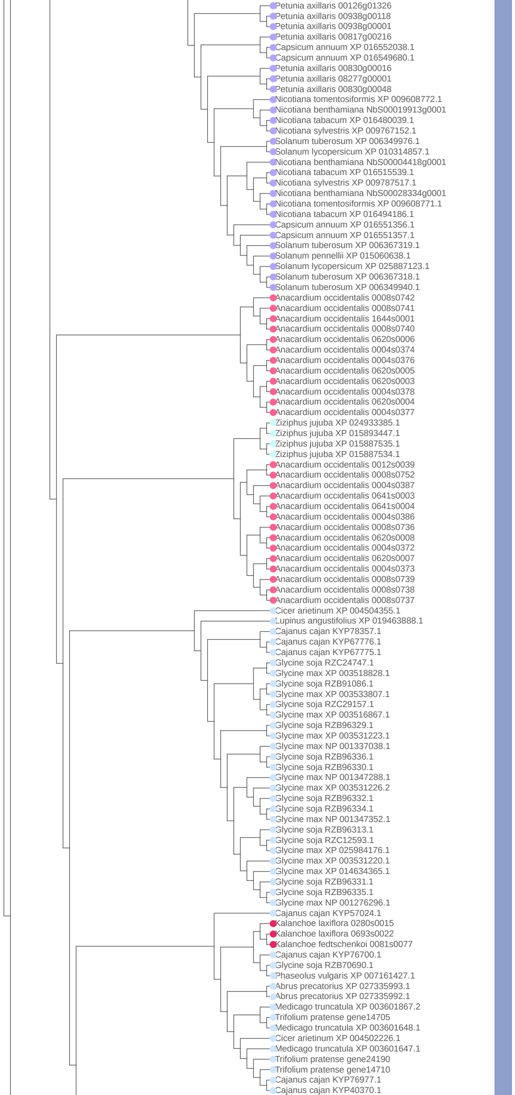
Petunia axillaris 00126g01326
Petunia axillaris 00938g00118
Petunia axillaris 00938g00001
Petunia axillaris 00817g00216
Capsicum annuum XP 016552038.1
Capsicum annuum XP 016549680.1
Petunia axillaris 00830g00016
Petunia axillaris 08277g00001
Petunia axillaris 00830g00048
Nicotiana tomentosiformis XP 009608772.1
Nicotiana benthamiana NbS00019913g0001
Nicotiana tabacum XP 016480039.1
Nicotiana sylvestris XP 009767152.1
Solanum tuberosum XP 006349976.1
Solanum lycopersicum XP 010314857.1
Nicotiana benthamiana NbS00004418g0001
Nicotiana tabacum XP 016515539.1
Nicotiana sylvestris XP 009787517.1
Nicotiana benthamiana NbS00028334g0001
Nicotiana tomentosiformis XP 009608771.1
Nicotiana tabacum XP 016494186.1
Capsicum annuum XP 016551356.1
Capsicum annuum XP 016551357.1
Solanum tuberosum XP 006367319.1
Solanum pennellii XP 015060638.1
Solanum lycopersicum XP 025887123.1
Solanum tuberosum XP 006367318.1
Solanum tuberosum XP 006349940.1
Anacardium occidentalis 0008s0742
Anacardium occidentalis 0008s0741
Anacardium occidentalis 1644s0001
Anacardium occidentalis 0008s0740
Anacardium occidentalis 0620s0006
Anacardium occidentalis 0004s0374
Anacardium occidentalis 0004s0376
Anacardium occidentalis 0620s0005
Anacardium occidentalis 0620s0003
Anacardium occidentalis 0004s0378
Anacardium occidentalis 0620s0004
Anacardium occidentalis 0004s0377
Ziziphus jujuba XP 024933385.1
Ziziphus jujuba XP 015893447.1
Ziziphus jujuba XP 015887535.1
Ziziphus jujuba XP 015887534.1
Anacardium occidentalis 0012s0039
Anacardium occidentalis 0008s0752
Anacardium occidentalis 0004s0387
Anacardium occidentalis 0641s0003
Anacardium occidentalis 0641s0004
Anacardium occidentalis 0004s0386
Anacardium occidentalis 0008s0736
Anacardium occidentalis 0620s0008
Anacardium occidentalis 0004s0372
Anacardium occidentalis 0620s0007
Anacardium occidentalis 0004s0373
Anacardium occidentalis 0008s0739
Anacardium occidentalis 0008s0738
Anacardium occidentalis 0008s0737
Cicer arietinum XP 004504355.1
Lupinus angustifolius XP 019463888.1
Cajanus cajan KYP78357.1
Cajanus cajan KYP67776.1
Cajanus cajan KYP67775.1
Glycine soja RZC24747.1
Glycine max XP 003518828.1
Glycine soja RZB91086.1
Glycine max XP 003533807.1
Glycine soja RZC29157.1
Glycine max XP 003516867.1
Glycine soja RZB96329.1
Glycine max XP 003531223.1
Glycine max NP 001337038.1
Glycine soja RZB96336.1
Glycine soja RZB96330.1
Glycine max NP 001347288.1
Glycine max XP 003531226.2
Glycine soja RZB96332.1
Glycine soja RZB96334.1
Glycine max NP 001347352.1
Glycine soja RZB96313.1
Glycine soja RZC12593.1
Glycine max XP 025984176.1
Glycine max XP 003531220.1
Glycine max XP 014634365.1
Glycine soja RZB96331.1
Glycine soja RZB96335.1
Glycine max NP 001276296.1
Cajanus cajan KYP57024.1
Kalanchoe laxiflora 0280s0015
Kalanchoe laxiflora 0693s0022
Kalanchoe fedtschenkoi 0081s0077
Cajanus cajan KYP76700.1
Glycine soja RZB70690.1
Phaseolus vulgaris XP 007161427.1
Abrus precatorius XP 027335993.1
Abrus precatorius XP 027335992.1
Medicago truncatula XP 003601867.2
Trifolium pratense gene14705
Medicago truncatula XP 003601648.1
Cicer arietinum XP 004502226.1
Medicago truncatula XP 003601647.1
Trifolium pratense gene24190
Trifolium pratense gene14710
Cajanus cajan KYP76977.1
Cajanus cajan KYP40370.1

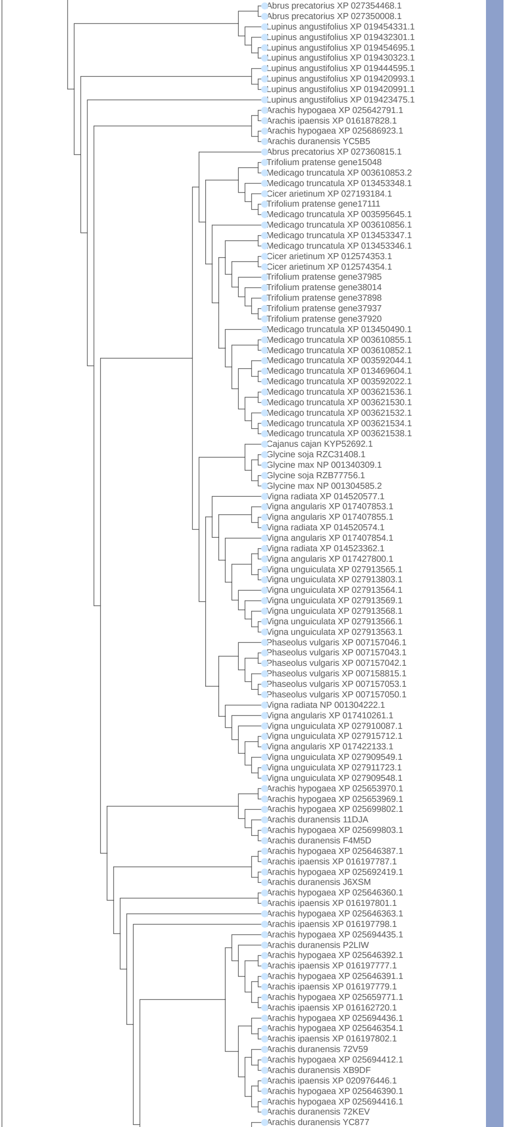

Abrus precatorius XP 027354468.1
Abrus precatorius XP 027350008.1
Lupinus angustifolius XP 019454331.1
Lupinus angustifolius XP 019432301.1
Lupinus angustifolius XP 019454695.1
Lupinus angustifolius XP 019430323.1
Lupinus angustifolius XP 019444595.1
Lupinus angustifolius XP 019420993.1
Lupinus angustifolius XP 019420991.1
Lupinus angustifolius XP 019423475.1
Arachis hypogaea XP 025642791.1
Arachis ipaensis XP 016187828.1
Arachis hypogaea XP 025686923.1
Arachis duranensis YC5B5
Abrus precatorius XP 027360815.1
Trifolium pratense gene15048
Medicago truncatula XP 003610853.2
Medicago truncatula XP 013453348.1
Cicer arietinum XP 027193184.1
Trifolium pratense gene17111
Medicago truncatula XP 003595645.1
Medicago truncatula XP 003610856.1
Medicago truncatula XP 013453347.1
Medicago truncatula XP 013453346.1
Cicer arietinum XP 012574353.1
Cicer arietinum XP 012574354.1
Trifolium pratense gene37985
Trifolium pratense gene38014
Trifolium pratense gene37898
Trifolium pratense gene37937
Trifolium pratense gene37920
Medicago truncatula XP 013450490.1
Medicago truncatula XP 003610855.1
Medicago truncatula XP 003610852.1
Medicago truncatula XP 003592044.1
Medicago truncatula XP 013469604.1
Medicago truncatula XP 003592022.1
Medicago truncatula XP 003621536.1
Medicago truncatula XP 003621530.1
Medicago truncatula XP 003621532.1
Medicago truncatula XP 003621534.1
Medicago truncatula XP 003621538.1
Cajanus cajan KYP52692.1
Glycine soja RZC31408.1
Glycine max NP 001340309.1
Glycine soja RZB77756.1
Glycine max NP 001304585.2
Vigna radiata XP 014520577.1
Vigna angularis XP 017407853.1
Vigna angularis XP 017407855.1
Vigna radiata XP 014520574.1
Vigna angularis XP 017407854.1
Vigna radiata XP 014523362.1
Vigna angularis XP 017427800.1
Vigna unguiculata XP 027913565.1
Vigna unguiculata XP 027913803.1
Vigna unguiculata XP 027913564.1
Vigna unguiculata XP 027913569.1
Vigna unguiculata XP 027913568.1
Vigna unguiculata XP 027913566.1
Vigna unguiculata XP 027913563.1
Phaseolus vulgaris XP 007157046.1
Phaseolus vulgaris XP 007157043.1
Phaseolus vulgaris XP 007157042.1
Phaseolus vulgaris XP 007158815.1
Phaseolus vulgaris XP 007157053.1
Phaseolus vulgaris XP 007157050.1
Vigna radiata NP 001304222.1
Vigna angularis XP 017410261.1
Vigna unguiculata XP 027910087.1
Vigna unguiculata XP 027915712.1
Vigna angularis XP 017422133.1
Vigna unguiculata XP 027909549.1
Vigna unguiculata XP 027911723.1
Vigna unguiculata XP 027909548.1
Arachis hypogaea XP 025653970.1
Arachis hypogaea XP 025653969.1
Arachis hypogaea XP 025699802.1
Arachis duranensis 11DJA
Arachis hypogaea XP 025699803.1
Arachis duranensis F4M5D
Arachis hypogaea XP 025646387.1
Arachis ipaensis XP 016197787.1
Arachis hypogaea XP 025692419.1
Arachis duranensis J6XSM
Arachis hypogaea XP 025646360.1
Arachis ipaensis XP 016197801.1
Arachis hypogaea XP 025646363.1
Arachis ipaensis XP 016197798.1
Arachis hypogaea XP 025694435.1
Arachis duranensis P2LIW
Arachis hypogaea XP 025646392.1
Arachis ipaensis XP 016197777.1
Arachis hypogaea XP 025646391.1
Arachis ipaensis XP 016197779.1
Arachis hypogaea XP 025659771.1
Arachis ipaensis XP 016162720.1
Arachis hypogaea XP 025694436.1
Arachis hypogaea XP 025646354.1
Arachis ipaensis XP 016197802.1
Arachis duranensis 72V59
Arachis hypogaea XP 025694412.1
Arachis duranensis XB9DF
Arachis ipaensis XP 020976446.1
Arachis hypogaea XP 025646390.1
Arachis hypogaea XP 025694416.1
Arachis duranensis 72KEV
Arachis duranensis YC877

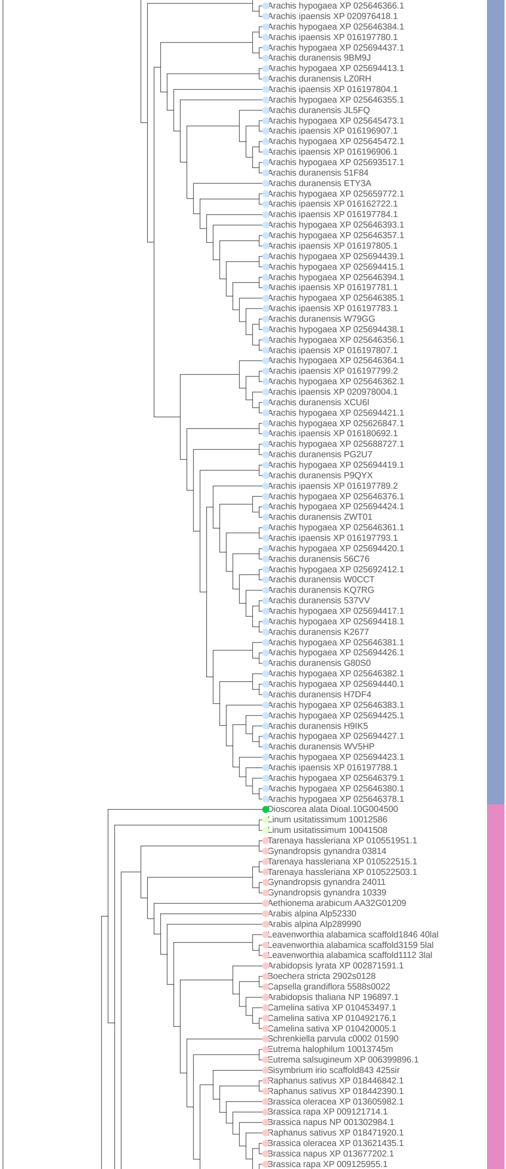
Arachis hypogaea XP 025646366.1
Arachis ipaensis XP 020976418.1
Arachis hypogaea XP 025646384.1
Arachis ipaensis XP 016197780.1
Arachis hypogaea XP 025694437.1
Arachis duranensis 9BM9J
Arachis hypogaea XP 025694413.1
Arachis duranensis LZ0RH
Arachis ipaensis XP 016197804.1
Arachis hypogaea XP 025646355.1
Arachis duranensis JL5FQ
Arachis hypogaea XP 025645473.1
Arachis ipaensis XP 016196907.1
Arachis hypogaea XP 025645472.1
Arachis ipaensis XP 016196906.1
Arachis hypogaea XP 025693517.1
Arachis duranensis 51F84
Arachis duranensis ETY3A
Arachis hypogaea XP 025659772.1
Arachis ipaensis XP 016162722.1
Arachis ipaensis XP 016197784.1
Arachis hypogaea XP 025646393.1
Arachis hypogaea XP 025646357.1
Arachis ipaensis XP 016197805.1
Arachis hypogaea XP 025694439.1
Arachis hypogaea XP 025694415.1
Arachis hypogaea XP 025646394.1
Arachis ipaensis XP 016197781.1
Arachis hypogaea XP 025646385.1
Arachis ipaensis XP 016197783.1
Arachis duranensis W79GG
Arachis hypogaea XP 025694438.1
Arachis hypogaea XP 025646356.1
Arachis ipaensis XP 016197807.1
Arachis hypogaea XP 025646364.1
Arachis ipaensis XP 016197799.2
Arachis hypogaea XP 025646362.1
Arachis ipaensis XP 020978004.1
Arachis duranensis XCU6I
Arachis hypogaea XP 025694421.1
Arachis hypogaea XP 025626847.1
Arachis ipaensis XP 016180692.1
Arachis hypogaea XP 025688727.1
Arachis duranensis PG2U7
Arachis hypogaea XP 025694419.1
Arachis duranensis P9QYX
Arachis ipaensis XP 016197789.2
Arachis hypogaea XP 025646376.1
Arachis hypogaea XP 025694424.1
Arachis duranensis ZWT01
Arachis hypogaea XP 025646361.1
Arachis ipaensis XP 016197793.1
Arachis hypogaea XP 025694420.1
Arachis duranensis 56C76
Arachis hypogaea XP 025692412.1
Arachis duranensis W0CCT
Arachis duranensis KQ7RG
Arachis duranensis 537VV
Arachis hypogaea XP 025694417.1
Arachis hypogaea XP 025694418.1
Arachis duranensis K2677
Arachis hypogaea XP 025646381.1
Arachis hypogaea XP 025694426.1
Arachis duranensis G80S0
Arachis hypogaea XP 025646382.1
Arachis hypogaea XP 025694440.1
Arachis duranensis H7DF4
Arachis hypogaea XP 025646383.1
Arachis hypogaea XP 025694425.1
Arachis duranensis H9IK5
Arachis hypogaea XP 025694427.1
Arachis duranensis WV5HP
Arachis hypogaea XP 025694423.1
Arachis ipaensis XP 016197788.1
Arachis hypogaea XP 025646379.1
Arachis hypogaea XP 025646380.1
Arachis hypogaea XP 025646378.1
Dioscorea alata Dioal.10G004500
Linum usitatissimum 10012586
Linum usitatissimum 10041508
Tarenaya hassleriana XP 010551951.1
Gynandropsis gynandra 03814
Tarenaya hassleriana XP 010522515.1
Tarenaya hassleriana XP 010522503.1
Gynandropsis gynandra 24011
Gynandropsis gynandra 10339
Aethionema arabicum AA32G01209
Arabis alpina Alp52330
Arabis alpina Alp289990
Leavenworthia alabamica scaffold1846 40lal
Leavenworthia alabamica scaffold3159 5lal
Leavenworthia alabamica scaffold1112 3lal
Arabidopsis lyrata XP 002871591.1
Boechera stricta 2902s0128
Capsella grandiflora 5588s0022
Arabidopsis thaliana NP 196897.1
Camelina sativa XP 010453497.1
Camelina sativa XP 010492176.1
Camelina sativa XP 010420005.1
Schrenkiella parvula c0002 01590
Eutrema halophilum 10013745m
Eutrema salsugineum XP 006399896.1
Sisymbrium irio scaffold843 425sir
Raphanus sativus XP 018446842.1
Raphanus sativus XP 018442390.1
Brassica oleracea XP 013605982.1
Brassica rapa XP 009121714.1
Brassica napus NP 001302984.1
Raphanus sativus XP 018471920.1
Brassica oleracea XP 013621435.1
Brassica napus XP 013677202.1
Brassica rapa XP 009125955.1

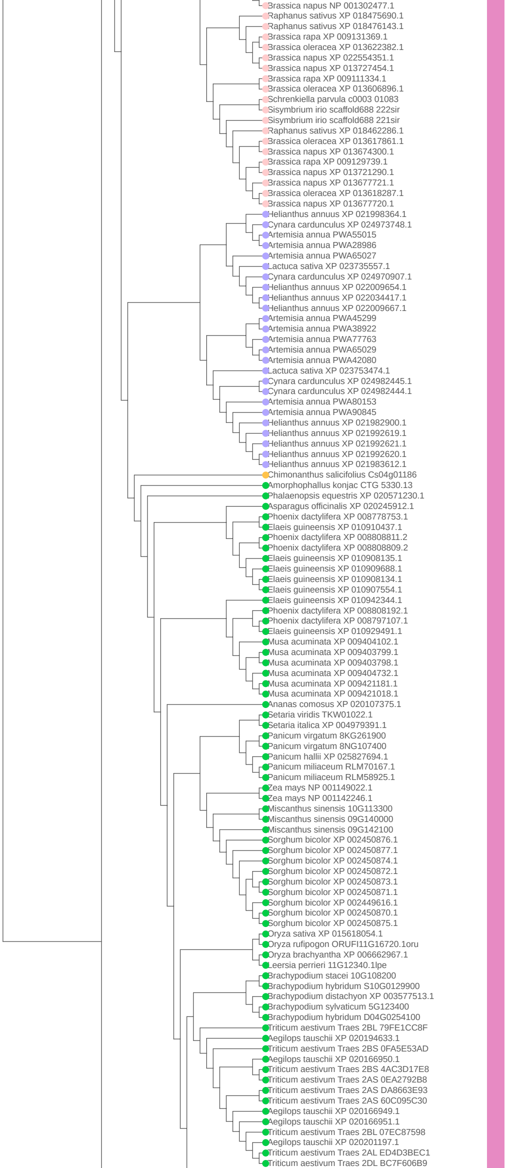
Brassica napus NP 001302477.1
Raphanus sativus XP 018475690.1
Raphanus sativus XP 018476143.1
Brassica rapa XP 009131369.1
Brassica oleracea XP 013622382.1
Brassica napus XP 022554351.1
Brassica napus XP 013727454.1
Brassica rapa XP 009111334.1
Brassica oleracea XP 013606896.1
Schrenkiella parvula c0003 01083
Sisymbrium irio scaffold688 222sir
Sisymbrium irio scaffold688 221sir
Raphanus sativus XP 018462286.1
Brassica oleracea XP 013617861.1
Brassica napus XP 013674300.1
Brassica rapa XP 009129739.1
Brassica napus XP 013721290.1
Brassica napus XP 013677721.1
Brassica oleracea XP 013618287.1
Brassica napus XP 013677720.1
Helianthus annuus XP 021998364.1
Cynara cardunculus XP 024973748.1
Artemisia annua PWA55015
Artemisia annua PWA28986
Artemisia annua PWA65027
Lactuca sativa XP 023735557.1
Cynara cardunculus XP 024970907.1
Helianthus annuus XP 022009654.1
Helianthus annuus XP 022034417.1
Helianthus annuus XP 022009667.1
Artemisia annua PWA45299
Artemisia annua PWA38922
Artemisia annua PWA77763
Artemisia annua PWA65029
Artemisia annua PWA42080
Lactuca sativa XP 023753474.1
Cynara cardunculus XP 024982445.1
Cynara cardunculus XP 024982444.1
Artemisia annua PWA80153
Artemisia annua PWA90845
Helianthus annuus XP 021982900.1
Helianthus annuus XP 021992619.1
Helianthus annuus XP 021992621.1
Helianthus annuus XP 021992620.1
Helianthus annuus XP 021983612.1
Chimonanthus salicifolius Cs04g01186
Amorphophallus konjac CTG 5330.13
Phalaenopsis equestris XP 020571230.1
Asparagus officinalis XP 020245912.1
Phoenix dactylifera XP 008778753.1
Elaeis guineensis XP 010910437.1
Phoenix dactylifera XP 008808811.2
Phoenix dactylifera XP 008808809.2
Elaeis guineensis XP 010908135.1
Elaeis guineensis XP 010909688.1
Elaeis guineensis XP 010908134.1
Elaeis guineensis XP 010907554.1
Elaeis guineensis XP 010942344.1
Phoenix dactylifera XP 008808192.1
Phoenix dactylifera XP 008797107.1
Elaeis guineensis XP 010929491.1
Musa acuminata XP 009404102.1
Musa acuminata XP 009403799.1
Musa acuminata XP 009403798.1
Musa acuminata XP 009404732.1
Musa acuminata XP 009421181.1
Musa acuminata XP 009421018.1
Ananas comosus XP 020107375.1
Setaria viridis TKW01022.1
Setaria italica XP 004979391.1
Panicum virgatum 8KG261900
Panicum virgatum 8NG107400
Panicum hallii XP 025827694.1
Panicum miliaceum RLM70167.1
Panicum miliaceum RLM58925.1
Zea mays NP 001149022.1
Zea mays NP 001142246.1
Miscanthus sinensis 10G113300
Miscanthus sinensis 09G140000
Miscanthus sinensis 09G142100
Sorghum bicolor XP 002450876.1
Sorghum bicolor XP 002450877.1
Sorghum bicolor XP 002450874.1
Sorghum bicolor XP 002450872.1
Sorghum bicolor XP 002450873.1
Sorghum bicolor XP 002450871.1
Sorghum bicolor XP 002449616.1
Sorghum bicolor XP 002450870.1
Sorghum bicolor XP 002450875.1
Oryza sativa XP 015618054.1
Oryza rufipogon ORUFI11G16720.1oru
Oryza brachyantha XP 006662967.1
Leersia perrieri 11G12340.1lpe
Brachypodium stacei 10G108200
Brachypodium hybridum S10G0129900
Brachypodium distachyon XP 003577513.1
Brachypodium sylvaticum 5G123400
Brachypodium hybridum D04G0254100
Triticum aestivum Traes 2BL 79FE1CC8F
Aegilops tauschii XP 020194633.1
Triticum aestivum Traes 2BS 0FA5E53AD
Aegilops tauschii XP 020166950.1
Triticum aestivum Traes 2BS 4AC3D17E8
Triticum aestivum Traes 2AS 0EA2792B8
Triticum aestivum Traes 2AS DA8663E93
Triticum aestivum Traes 2AS 60C095C30
Aegilops tauschii XP 020166949.1
Aegilops tauschii XP 020166951.1
Triticum aestivum Traes 2BL 07EC87598
Aegilops tauschii XP 020201197.1
Triticum aestivum Traes 2AL ED4D3BEC1
Triticum aestivum Traes 2DL BC7F606B9

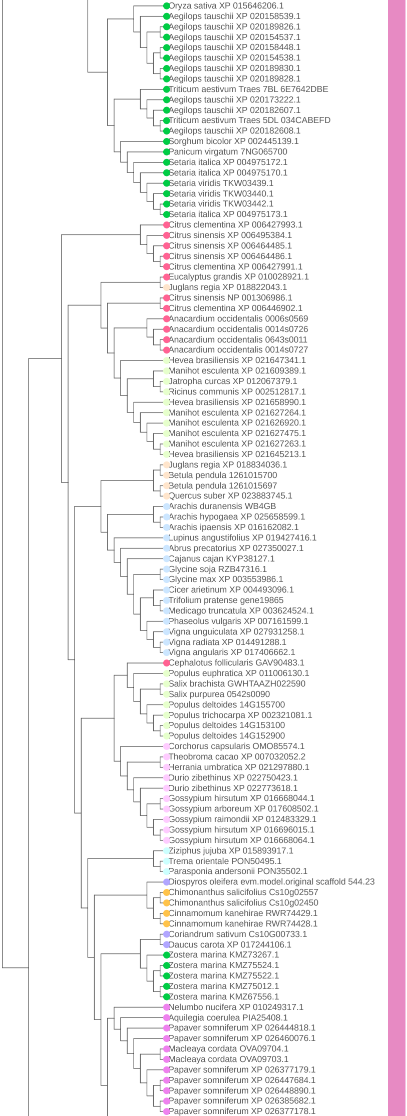
Oryza sativa XP 015646206.1
Aegilops tauschii XP 020158539.1
Aegilops tauschii XP 020189826.1
Aegilops tauschii XP 020154537.1
Aegilops tauschii XP 020158448.1
Aegilops tauschii XP 020154538.1
Aegilops tauschii XP 020189830.1
Aegilops tauschii XP 020189828.1
Triticum aestivum Traes 7BL 6E7642DBE
Aegilops tauschii XP 020173222.1
Aegilops tauschii XP 020182607.1
Triticum aestivum Traes 5DL 034CABEFD
Aegilops tauschii XP 020182608.1
Sorghum bicolor XP 002445139.1
Panicum virgatum 7NG065700
Setaria italica XP 004975172.1
Setaria italica XP 004975170.1
Setaria viridis TKW03439.1
Setaria viridis TKW03440.1
Setaria viridis TKW03442.1
Setaria italica XP 004975173.1
Citrus clementina XP 006427993.1
Citrus sinensis XP 006495384.1
Citrus sinensis XP 006464485.1
Citrus sinensis XP 006464486.1
Citrus clementina XP 006427991.1
Eucalyptus grandis XP 010028921.1
Juglans regia XP 018822043.1
Citrus sinensis NP 001306986.1
Citrus clementina XP 006446902.1
Anacardium occidentalis 0006s0569
Anacardium occidentalis 0014s0726
Anacardium occidentalis 0643s0011
Anacardium occidentalis 0014s0727
Hevea brasiliensis XP 021647341.1
Manihot esculenta XP 021609389.1
Jatropha curcas XP 012067379.1
Ricinus communis XP 002512817.1
Hevea brasiliensis XP 021658990.1
Manihot esculenta XP 021627264.1
Manihot esculenta XP 021626920.1
Manihot esculenta XP 021627475.1
Manihot esculenta XP 021627263.1
Hevea brasiliensis XP 021645213.1
Juglans regia XP 018834036.1
Betula pendula 1261015700
Betula pendula 1261015697
Quercus suber XP 023883745.1
Arachis duranensis WB4GB
Arachis hypogaea XP 025658599.1
Arachis ipaensis XP 016162082.1
Lupinus angustifolius XP 019427416.1
Abrus precatorius XP 027350027.1
Cajanus cajan KYP38127.1
Glycine soja RZB47316.1
Glycine max XP 003553986.1
Cicer arietinum XP 004493096.1
Trifolium pratense gene19865
Medicago truncatula XP 003624524.1
Phaseolus vulgaris XP 007161599.1
Vigna unguiculata XP 027931258.1
Vigna radiata XP 014491288.1
Vigna angularis XP 017406662.1
Cephalotus follicularis GAV90483.1
Populus euphratica XP 011006130.1
Salix brachista GWHTAAZH022590
Salix purpurea 0542s0090
Populus deltoides 14G155700
Populus trichocarpa XP 002321081.1
Populus deltoides 14G153100
Populus deltoides 14G152900
Corchorus capsularis OMO85574.1
Theobroma cacao XP 007032052.2
Herrania umbratica XP 021297880.1
Durio zibethinus XP 022750423.1
Durio zibethinus XP 022773618.1
Gossypium hirsutum XP 016668044.1
Gossypium arboreum XP 017608502.1
Gossypium raimondii XP 012483329.1
Gossypium hirsutum XP 016696015.1
Gossypium hirsutum XP 016668064.1
Ziziphus jujuba XP 015893917.1
Trema orientale PON50495.1
Parasponia andersonii PON35502.1
Diospyros oleifera evm.model.original scaffold 544.23
Chimonanthus salicifolius Cs10g02557
Chimonanthus salicifolius Cs10g02450
Cinnamomum kanehirae RWR74429.1
Cinnamomum kanehirae RWR74428.1
Coriandrum sativum Cs10G00733.1
Daucus carota XP 017244106.1
Zostera marina KMZ73267.1
Zostera marina KMZ75524.1
Zostera marina KMZ75522.1
Zostera marina KMZ75012.1
Zostera marina KMZ67556.1
Nelumbo nucifera XP 010249317.1
Aquilegia coerulea PIA25408.1
Papaver somniferum XP 026444818.1
Papaver somniferum XP 026460076.1
Macleaya cordata OVA09704.1
Macleaya cordata OVA09703.1
Papaver somniferum XP 026377179.1
Papaver somniferum XP 026447684.1
Papaver somniferum XP 026448890.1
Papaver somniferum XP 026385682.1
Papaver somniferum XP 026377178.1

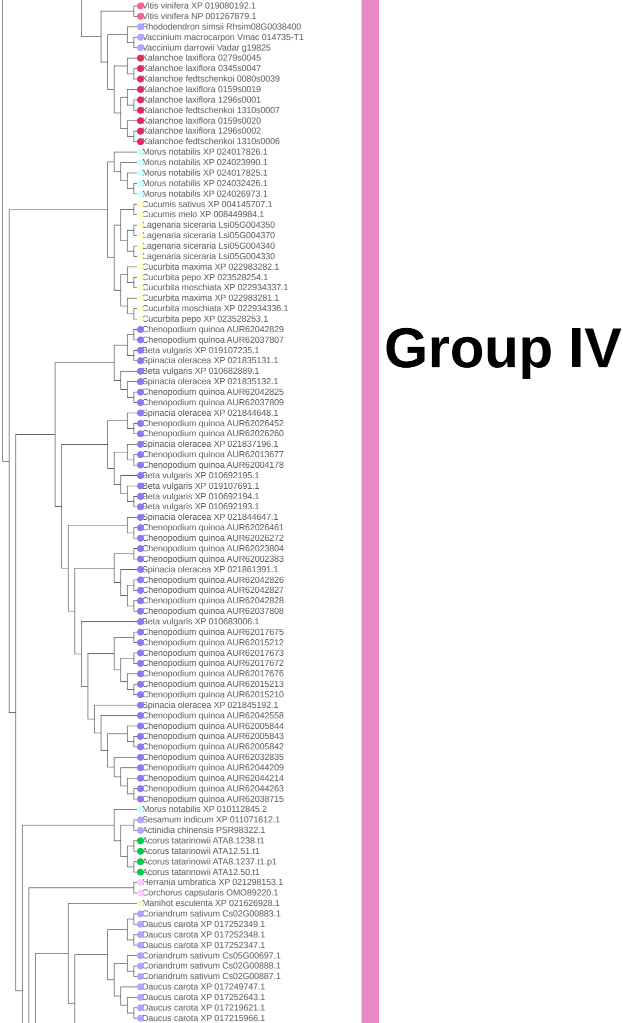
Vitis vinifera XP 019080192.1
Vitis vinifera NP 001267879.1
Rhododendron simsii Rhsim08G0038400
Vaccinium macrocarpon Vmac 014735-T1
Vaccinium darrowii Vadar g19825
Kalanchoe laxiflora 0279s0045
Kalanchoe laxiflora 0345s0047
Kalanchoe fedtschenkoi 0080s0039
Kalanchoe laxiflora 0159s0019
Kalanchoe laxiflora 1296s0001
Kalanchoe fedtschenkoi 1310s0007
Kalanchoe laxiflora 0159s0020
Kalanchoe laxiflora 1296s0002
Kalanchoe fedtschenkoi 1310s0006
Morus notabilis XP 024017826.1
Morus notabilis XP 024023990.1
Morus notabilis XP 024017825.1
Morus notabilis XP 024032426.1
Morus notabilis XP 024026973.1
Cucumis sativus XP 004145707.1
Cucumis melo XP 008449984.1
Lagenaria siceraria Lsi05G004350
Lagenaria siceraria Lsi05G004370
Lagenaria siceraria Lsi05G004340
Lagenaria siceraria Lsi05G004330
Cucurbita maxima XP 022983282.1
Cucurbita pepo XP 023528254.1
Cucurbita moschiata XP 022934337.1
Cucurbita maxima XP 022983281.1
Cucurbita moschiata XP 022934336.1
Cucurbita pepo XP 023528253.1
Chenopodium quinoa AUR62042829
Chenopodium quinoa AUR62037807
Beta vulgaris XP 019107235.1
Spinacia oleracea XP 021835131.1
Beta vulgaris XP 010682889.1
Spinacia oleracea XP 021835132.1
Chenopodium quinoa AUR62042825
Chenopodium quinoa AUR62037809
Spinacia oleracea XP 021844648.1
Chenopodium quinoa AUR62026452
Chenopodium quinoa AUR62026260
Spinacia oleracea XP 021837196.1
Chenopodium quinoa AUR62013677
Chenopodium quinoa AUR62004178
Beta vulgaris XP 010692195.1
Beta vulgaris XP 019107691.1
Beta vulgaris XP 010692194.1
Beta vulgaris XP 010692193.1
Spinacia oleracea XP 021844647.1
Chenopodium quinoa AUR62026461
Chenopodium quinoa AUR62026272
Chenopodium quinoa AUR62023804
Chenopodium quinoa AUR62002383
Spinacia oleracea XP 021861391.1
Chenopodium quinoa AUR62042826
Chenopodium quinoa AUR62042827
Chenopodium quinoa AUR62042828
Chenopodium quinoa AUR62037808
Beta vulgaris XP 010683006.1
Chenopodium quinoa AUR62017675
Chenopodium quinoa AUR62015212
Chenopodium quinoa AUR62017673
Chenopodium quinoa AUR62017672
Chenopodium quinoa AUR62017676
Chenopodium quinoa AUR62015213
Chenopodium quinoa AUR62015210
Spinacia oleracea XP 021845192.1
Chenopodium quinoa AUR62042558
Chenopodium quinoa AUR62005844
Chenopodium quinoa AUR62005843
Chenopodium quinoa AUR62005842
Chenopodium quinoa AUR62032835
Chenopodium quinoa AUR62044209
Chenopodium quinoa AUR62044214
Chenopodium quinoa AUR62044263
Chenopodium quinoa AUR62038715
Morus notabilis XP 010112845.2
Sesamum indicum XP 011071612.1
Actinidia chinensis PSR98322.1
Acorus tatarinowii ATA8.1238.t1
Acorus tatarinowii ATA12.51.t1
Acorus tatarinowii ATA8.1237.t1.p1
Acorus tatarinowii ATA12.50.t1
Herrania umbratica XP 021298153.1
Corchorus capsularis OMO89220.1
Manihot esculenta XP 021626928.1
Coriandrum sativum Cs02G00883.1
Daucus carota XP 017252349.1
Daucus carota XP 017252348.1
Daucus carota XP 017252347.1
Coriandrum sativum Cs05G00697.1
Coriandrum sativum Cs02G00888.1
Coriandrum sativum Cs02G00887.1
Daucus carota XP 017249747.1
Daucus carota XP 017252643.1
Daucus carota XP 017219621.1
Daucus carota XP 017215966.1
Group IV

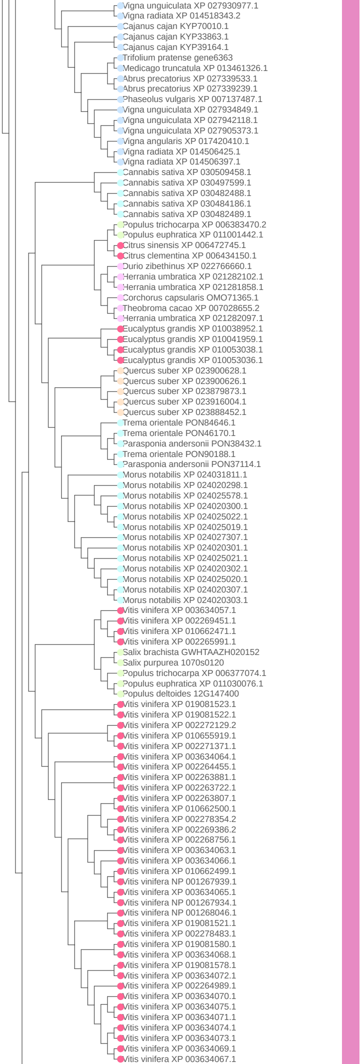
Vigna unguiculata XP 027930977.1
Vigna radiata XP 014518343.2
Cajanus cajan KYP70010.1
Cajanus cajan KYP33863.1
Cajanus cajan KYP39164.1
Trifolium pratense gene6363
Medicago truncatula XP 013461326.1
Abrus precatorius XP 027339533.1
Abrus precatorius XP 027339239.1
Phaseolus vulgaris XP 007137487.1
Vigna unguiculata XP 027934849.1
Vigna unguiculata XP 027942118.1
Vigna unguiculata XP 027905373.1
Vigna angularis XP 017420410.1
Vigna radiata XP 014506425.1
Vigna radiata XP 014506397.1
Cannabis sativa XP 030509458.1
Cannabis sativa XP 030497599.1
Cannabis sativa XP 030482488.1
Cannabis sativa XP 030484186.1
Cannabis sativa XP 030482489.1
Populus trichocarpa XP 006383470.2
Populus euphratica XP 011001442.1
Citrus sinensis XP 006472745.1
Citrus clementina XP 006434150.1
Durio zibethinus XP 022766660.1
Herrania umbratica XP 021282102.1
Herrania umbratica XP 021281858.1
Corchorus capsularis OMO71365.1
Theobroma cacao XP 007028655.2
Herrania umbratica XP 021282097.1
Eucalyptus grandis XP 010038952.1
Eucalyptus grandis XP 010041959.1
Eucalyptus grandis XP 010053038.1
Eucalyptus grandis XP 010053036.1
Quercus suber XP 023900628.1
Quercus suber XP 023900626.1
Quercus suber XP 023879873.1
Quercus suber XP 023916004.1
Quercus suber XP 023888452.1
Trema orientale PON84646.1
Trema orientale PON46170.1
Parasponia andersonii PON38432.1
Trema orientale PON90188.1
Parasponia andersonii PON37114.1
Morus notabilis XP 024031811.1
Morus notabilis XP 024020298.1
Morus notabilis XP 024025578.1
Morus notabilis XP 024020300.1
Morus notabilis XP 024025022.1
Morus notabilis XP 024025019.1
Morus notabilis XP 024027307.1
Morus notabilis XP 024020301.1
Morus notabilis XP 024025021.1
Morus notabilis XP 024020302.1
Morus notabilis XP 024025020.1
Morus notabilis XP 024020307.1
Morus notabilis XP 024020303.1
Vitis vinifera XP 003634057.1
Vitis vinifera XP 002269451.1
Vitis vinifera XP 010662471.1
Vitis vinifera XP 002265991.1
Salix brachista GWHTAAZH020152
Salix purpurea 1070s0120
Populus trichocarpa XP 006377074.1
Populus euphratica XP 011030076.1
Populus deltoides 12G147400
Vitis vinifera XP 019081523.1
Vitis vinifera XP 019081522.1
Vitis vinifera XP 002272129.2
Vitis vinifera XP 010655919.1
Vitis vinifera XP 002271371.1
Vitis vinifera XP 003634064.1
Vitis vinifera XP 002264455.1
Vitis vinifera XP 002263881.1
Vitis vinifera XP 002263722.1
Vitis vinifera XP 002263807.1
Vitis vinifera XP 010662500.1
Vitis vinifera XP 002278354.2
Vitis vinifera XP 002269386.2
Vitis vinifera XP 002268756.1
Vitis vinifera XP 003634063.1
Vitis vinifera XP 003634066.1
Vitis vinifera XP 010662499.1
Vitis vinifera NP 001267939.1
Vitis vinifera XP 003634065.1
Vitis vinifera NP 001267934.1
Vitis vinifera NP 001268046.1
Vitis vinifera XP 019081521.1
Vitis vinifera XP 002278483.1
Vitis vinifera XP 019081580.1
Vitis vinifera XP 003634068.1
Vitis vinifera XP 019081578.1
Vitis vinifera XP 003634072.1
Vitis vinifera XP 002264989.1
Vitis vinifera XP 003634070.1
Vitis vinifera XP 003634075.1
Vitis vinifera XP 003634071.1
Vitis vinifera XP 003634074.1
Vitis vinifera XP 003634073.1
Vitis vinifera XP 003634069.1
Vitis vinifera XP 003634067.1

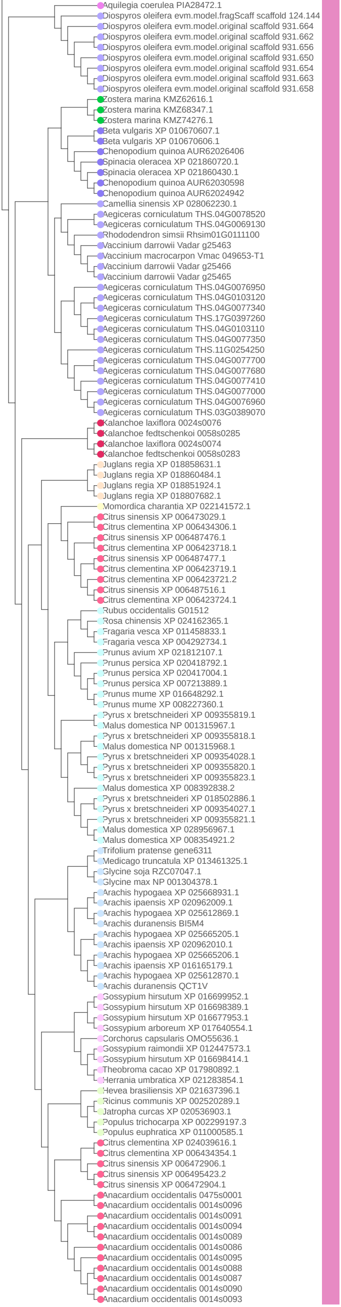

Aquilegia coerulea PIA28472.1
Diospyros oleifera evm.model.fragScaff scaffold 124.144
Diospyros oleifera evm.model.original scaffold 931.664
Diospyros oleifera evm.model.original scaffold 931.662
Diospyros oleifera evm.model.original scaffold 931.656
Diospyros oleifera evm.model.original scaffold 931.650
Diospyros oleifera evm.model.original scaffold 931.654
Diospyros oleifera evm.model.original scaffold 931.663
Diospyros oleifera evm.model.original scaffold 931.658
Zostera marina KMZ62616.1
Zostera marina KMZ68347.1
Zostera marina KMZ74276.1
Beta vulgaris XP 010670607.1
Beta vulgaris XP 010670606.1
Chenopodium quinoa AUR62026406
Spinacia oleracea XP 021860720.1
Spinacia oleracea XP 021860430.1
Chenopodium quinoa AUR62030598
Chenopodium quinoa AUR62024942
Camellia sinensis XP 028062230.1
Aegiceras corniculatum THS.04G0078520
Aegiceras corniculatum THS.04G0069130
Rhododendron simsii Rhsim01G0111100
Vaccinium darrowii Vadar g25463
Vaccinium macrocarpon Vmac 049653-T1
Vaccinium darrowii Vadar g25466
Vaccinium darrowii Vadar g25465
Aegiceras corniculatum THS.04G0076950
Aegiceras corniculatum THS.04G0103120
Aegiceras corniculatum THS.04G0077340
Aegiceras corniculatum THS.17G0397260
Aegiceras corniculatum THS.04G0103110
Aegiceras corniculatum THS.04G0077350
Aegiceras corniculatum THS.11G0254250
Aegiceras corniculatum THS.04G0077700
Aegiceras corniculatum THS.04G0077680
Aegiceras corniculatum THS.04G0077410
Aegiceras corniculatum THS.04G0077000
Aegiceras corniculatum THS.04G0076960
Aegiceras corniculatum THS.03G0389070
Kalanchoe laxiflora 0024s0076
Kalanchoe fedtschenkoi 0058s0285
Kalanchoe laxiflora 0024s0074
Kalanchoe fedtschenkoi 0058s0283
Juglans regia XP 018858631.1
Juglans regia XP 018860484.1
Juglans regia XP 018851924.1
Juglans regia XP 018807682.1
Momordica charantia XP 022141572.1
Citrus sinensis XP 006473029.1
Citrus clementina XP 006434306.1
Citrus sinensis XP 006487476.1
Citrus clementina XP 006423718.1
Citrus sinensis XP 006487477.1
Citrus clementina XP 006423719.1
Citrus clementina XP 006423721.2
Citrus sinensis XP 006487516.1
Citrus clementina XP 006423724.1
Rubus occidentalis G01512
Rosa chinensis XP 024162365.1
Fragaria vesca XP 011458833.1
Fragaria vesca XP 004292734.1
Prunus avium XP 021812107.1
Prunus persica XP 020418792.1
Prunus persica XP 020417004.1
Prunus persica XP 007213889.1
Prunus mume XP 016648292.1
Prunus mume XP 008227360.1
Pyrus x bretschneideri XP 009355819.1
Malus domestica NP 001315967.1
Pyrus x bretschneideri XP 009355818.1
Malus domestica NP 001315968.1
Pyrus x bretschneideri XP 009354028.1
Pyrus x bretschneideri XP 009355820.1
Pyrus x bretschneideri XP 009355823.1
Malus domestica XP 008392838.2
Pyrus x bretschneideri XP 018502886.1
Pyrus x bretschneideri XP 009354027.1
Pyrus x bretschneideri XP 009355821.1
Malus domestica XP 028956967.1
Malus domestica XP 008354921.2
Trifolium pratense gene6311
Medicago truncatula XP 013461325.1
Glycine soja RZC07047.1
Glycine max NP 001304378.1
Arachis hypogaea XP 025668931.1
Arachis ipaensis XP 020962009.1
Arachis hypogaea XP 025612869.1
Arachis duranensis BI5M4
Arachis hypogaea XP 025665205.1
Arachis ipaensis XP 020962010.1
Arachis hypogaea XP 025665206.1
Arachis ipaensis XP 016165179.1
Arachis hypogaea XP 025612870.1
Arachis duranensis QCT1V
Gossypium hirsutum XP 016699952.1
Gossypium hirsutum XP 016698389.1
Gossypium hirsutum XP 016677953.1
Gossypium arboreum XP 017640554.1
Corchorus capsularis OMO55636.1
Gossypium raimondii XP 012447573.1
Gossypium hirsutum XP 016698414.1
Theobroma cacao XP 017980892.1
Herrania umbratica XP 021283854.1
Hevea brasiliensis XP 021637396.1
Ricinus communis XP 002520289.1
Jatropha curcas XP 020536903.1
Populus trichocarpa XP 002299197.3
Populus euphratica XP 011000585.1
Citrus clementina XP 024039616.1
Citrus clementina XP 006434354.1
Citrus sinensis XP 006472906.1
Citrus sinensis XP 006495423.2
Citrus sinensis XP 006472904.1
Anacardium occidentalis 0475s0001
Anacardium occidentalis 0014s0096
Anacardium occidentalis 0014s0091
Anacardium occidentalis 0014s0094
Anacardium occidentalis 0014s0089
Anacardium occidentalis 0014s0086
Anacardium occidentalis 0014s0095
Anacardium occidentalis 0014s0088
Anacardium occidentalis 0014s0087
Anacardium occidentalis 0014s0090
Anacardium occidentalis 0014s0093
